# Synergistic Anticancer Effects of Resveratrol and Carboplatin in Y79 Retinoblastoma Cells: Mechanistic Insights into Apoptosis, G2/M Arrest, and ROS-Dependent Mitochondrial Dysfunction

**DOI:** 10.3390/ijms27083473

**Published:** 2026-04-13

**Authors:** Aydın Maçin, Erkan Duman, İlhan Özdemir, Mehmet Cudi Tuncer

**Affiliations:** 1 Department of Ophthalmology, Diyarbakır Private Batı Hospital, 21100 Diyarbakır, Turkey; draydinmacin@outlook.com; 2 Department of Ophthalmology, WestEye Private Hospital, Erbil 44001, Iraq; ophthalmo48@outlook.com; 3 Department of Histology and Embryology, Faculty of Medicine, Kahramanmaraş Sütçü İmam University, 46040 Kahramanmaraş, Turkey; ilhanozdemir25@yandex.com; 4 Department of Anatomy, Faculty of Medicine, Dicle University, 21280 Diyarbakır, Turkey

**Keywords:** retinoblastoma, Y79, resveratrol, carboplatin, apoptosis, synergy

## Abstract

This study aimed to investigate the effects of resveratrol (RES) and carboplatin (CPT), alone and in combination, on cell viability, apoptosis, cell cycle progression, mitochondrial function, and oxidative stress in Y79 retinoblastoma (RB) cells. Particular emphasis was placed on evaluating the synergistic potential of the combination and elucidating the interconnected molecular mechanisms underlying its anticancer effects. Y79 cells were treated with RES, CPT, and their combinations. Cell viability and synergy were assessed using the MTT assay and combination index (CI) analysis. Apoptosis (annexin V/PI), cell cycle distribution (propidium iodide (PI) staining), intracellular ROS production (DCFH-DA), and mitochondrial membrane potential (JC-1) were evaluated by flow cytometry. ROS dependency was further examined using N-acetylcysteine (NAC) pretreatment. Expression levels of apoptosis- and cell cycle-related genes (*BAX*, *BCL-2*, *CASP3*, *CASP9*, *CCNB1*, and *CDK1*) were analyzed by RT-qPCR. Cytoskeletal alterations were assessed by immunocytochemistry. In addition, the antitumor effects of the combination were validated in a three-dimensional (3D) tumor spheroid model. RES and CPT reduced cell viability in a dose- and time-dependent manner and demonstrated synergistic effects (CI < 1) at selected concentrations. Combination treatment significantly increased apoptosis, induced G2/M phase arrest, enhanced ROS accumulation, and promoted mitochondrial depolarization compared with single-agent treatments. NAC pretreatment attenuated ROS generation and partially restored cell viability, supporting a contributory role of oxidative stress in combination-induced cytotoxicity. At the transcriptional level, the RES + CPT combination significantly increased the *BAX*/*BCL-2* ratio and upregulated *CASP3* and *CASP9* expression, while downregulating *CCNB1* and *CDK1*, consistent with mitochondrial apoptotic activation and G2/M arrest. Immunocytochemical analysis revealed pronounced cytoskeletal disruption and apoptotic morphology in the combination group. Importantly, in the 3D spheroid model, co-treatment markedly reduced spheroid size and viability and enhanced cell death compared with monotherapies. The combination of RES and CPT exerts a synergistic anticancer effect in Y79 RB cells through coordinated mechanisms involving ROS accumulation, mitochondrial dysfunction, caspase activation, and G2/M phase arrest. The attenuation of cytotoxicity by NAC and the validation of efficacy in a 3D tumor spheroid model strengthen the mechanistic relevance of these findings. These results support further preclinical investigation of this combination strategy in in vivo models and normal retinal cell systems.

## 1. Introduction

RB is the most common primary intraocular malignancy in childhood and results from mutations in the retinoblastoma 1 gene (RB1) tumor suppressor gene [1]. Current treatment protocols include intra-arterial, intravenous, or intravitreal chemotherapy, as well as radiotherapy and enucleation, but recurrence rates in high-risk patients remain a significant clinical problem [2,3]. Furthermore, the chemotherapeutic agents used (platinum derivatives, etoposide, vincristine, etc.) have serious side effects such as increased risk of secondary malignancy, hearing loss, and neurotoxicity [4]. Therefore, new therapeutic strategies are needed to improve the efficacy and toxicity profiles of current treatments. Combination therapies offer the potential to increase treatment efficacy through synergistic effects while simultaneously reducing drug dosage and associated toxicity [5].

Natural products constitute a rich source for the discovery of new anticancer agents. RES (3,5,4′-trihydroxy stilbene) is a polyphenolic stilbene found in plants such as grapes, peanuts, and mulberries and has a broad spectrum of pharmacological activity [6]. In vitro and in vivo studies have revealed the antioxidant, anti-inflammatory, and anticancer properties of RES [7]. Anticancer mechanisms include arresting various phases of the cell cycle (especially G1/S and G2/M transitions), inducing apoptosis, inhibiting angiogenesis, and suppressing metastasis [8,9]. In particular, RES has been reported to selectively promote death in cancer cells by triggering reactive oxygen species (ROS)-mediated mitochondrial dysfunction and activating the intrinsic (mitochondrial) apoptotic pathway [10].

CPT is a second-generation platinum compound that inhibits replication and transcription by forming cross-links to DNA, thereby inducing cell death [11]. In RB, CPT is one of the cornerstones of intra-arterial chemotherapy protocols [3]. However, resistance and systemic toxicity that develop with its use alone are significant obstacles limiting its effectiveness [12]. In this context, the combination of CPT with natural compounds stands out as a promising approach to break resistance and improve the therapeutic index. The synergistic effect between RES and platinum-based agents has been demonstrated in various cancer models such as colorectal, ovarian, lung, and bladder cancer [12,13,14,15]. Furthermore, RES has been reported to inhibit the migration of MDA231 cells by reversing TGF-β1-induced EMT [16]. However, comprehensive data on the effects of the combination of RES and CPT on RB, particularly the aggressive Y79 cell line, and the underlying molecular mechanisms are limited.

Despite growing evidence supporting the anticancer properties of RES and the clinical relevance of CPT in RB therapy, comprehensive data regarding their combined effects in Y79 cells and the underlying molecular mechanisms remain limited. We hypothesized that RES enhances the cytotoxic and apoptotic effects of CPT through coordinated mechanisms involving G2/M cell cycle arrest, mitochondrial dysfunction, and modulation of intracellular ROS signaling. To test this hypothesis, we systematically evaluated the antiproliferative and pro-apoptotic effects of these agents using complementary approaches, including cell viability and synergy analysis, flow cytometric assessment of apoptosis, cell cycle distribution, ROS production, and mitochondrial membrane potential, RT-qPCR analysis of apoptosis- and cell cycle-related genes, and immunocytochemical evaluation of cytoskeletal alterations. The biological relevance of the combination was further examined in a three-dimensional (3D) tumor spheroid model, and the functional contribution of oxidative stress was investigated using NAC pretreatment. This integrated strategy was designed to provide mechanistic insight into the interaction between RES and CPT and to support further preclinical investigation of this combination in RB.

## 2. Results

### 2.1. Dose- and Time-Dependent Cytotoxic Effects of RES and CPT in Y79 Retinoblastoma Cells (MTT Assay)

**Figure 1:**
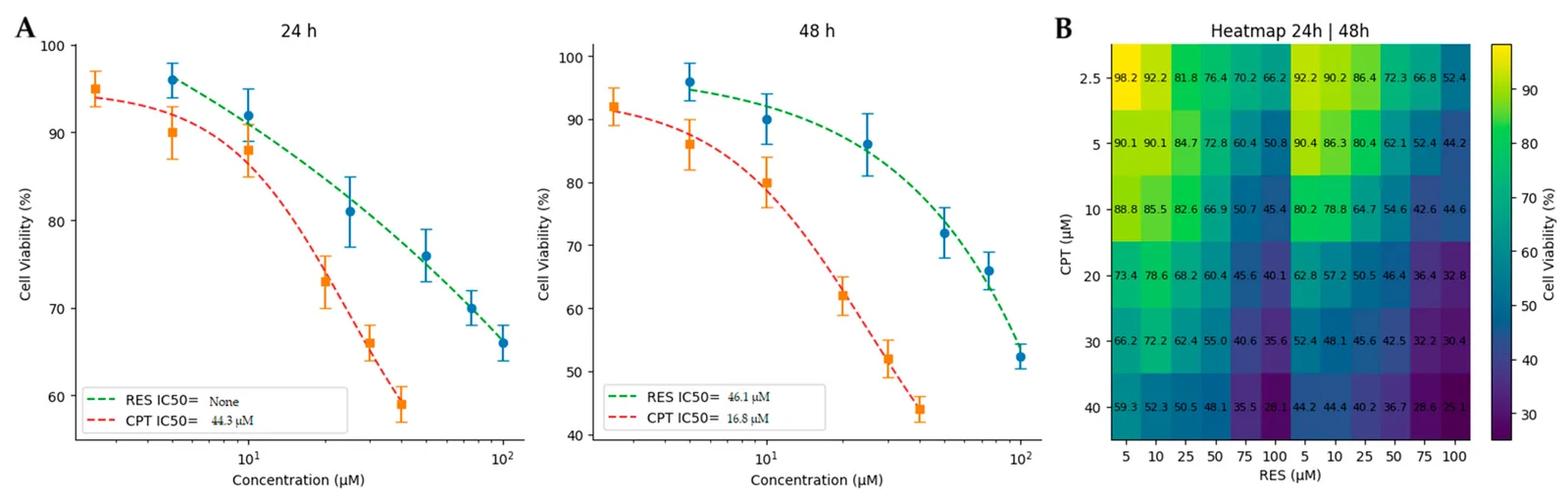
(**A**) Dose- and time-dependent effects of RES and CPT monotherapies on Y-79 cell viability assessed by MTT assay after 24 h and 48 h treatment. Cell viability is expressed as a percentage relative to the untreated control (set to 100%). A nonlinear regression model (log[inhibitor] vs. normalized response, variable slope) was applied to generate sigmoidal dose–response curves. Both RES and CPT reduced cell viability in a concentration and time-dependent manner. The calculated IC_50_ values at 24 h and 48 h were: RES 51.1 µM (24 h) and 46.1 µM (48 h); CPT 44.3 µM (24 h) and 16.8 µM (48 h). Data are presented as mean ± SD of three independent experiments (n = 3). (**B**) Heatmap representation of cell viability following treatment with RES, CPT, and their combinations at indicated concentrations for 24 h and 48 h. Percentage values correspond to mean viability relative to the control. Combined treatments (RES + CPT) resulted in a greater reduction in viability compared to single-agent treatments, particularly at higher concentrations and longer exposure times.

The cytotoxic effects of RES and CPT on Y79 retinoblastoma cells were evaluated by MTT analysis after 24 and 48 h of application. Both agents reduced cell viability in a dose- and time-dependent manner (Figure 1A).

In RES monotherapy, a limited decrease in cell viability was observed at low doses (5–10 µM) after 24 h of application, while a more significant decrease occurred at concentrations of 25 µM and above. At 48 h, the cytotoxic effect became more pronounced, and cell viability was significantly reduced, particularly in the 50–100 µM range. Nonlinear regression analysis yielded IC_50_ values of 51.1 µM at 24 h and 46.5 µM at 48 h for RES.

Similarly, a dose- and time-dependent cytotoxic effect was observed in CPT monotherapy. In the 24 h application, concentrations of 10 µM and above significantly reduced cell viability, while this effect was further increased in the 48 h application. IC_50_ values for CPT were calculated as 16.8 µM at 24 h and 14.3 µM at 48 h. These results indicate that CPT has a higher cytotoxic potential compared to RES.

The effects of combination therapy were visualized using heatmap analysis (Figure 1B). In both the 24 h and 48 h applications, it was observed that combinations of RES and CPT significantly reduced cell viability compared to single applications. This effect was particularly pronounced in medium- and high-dose combinations, and it is noteworthy that the cytotoxic effect increased with increasing time.

### 2.2. Mathematical Validation of RES–CPT Synergy in Y79 Cells Using CI and Bliss Models

**Figure 2:**
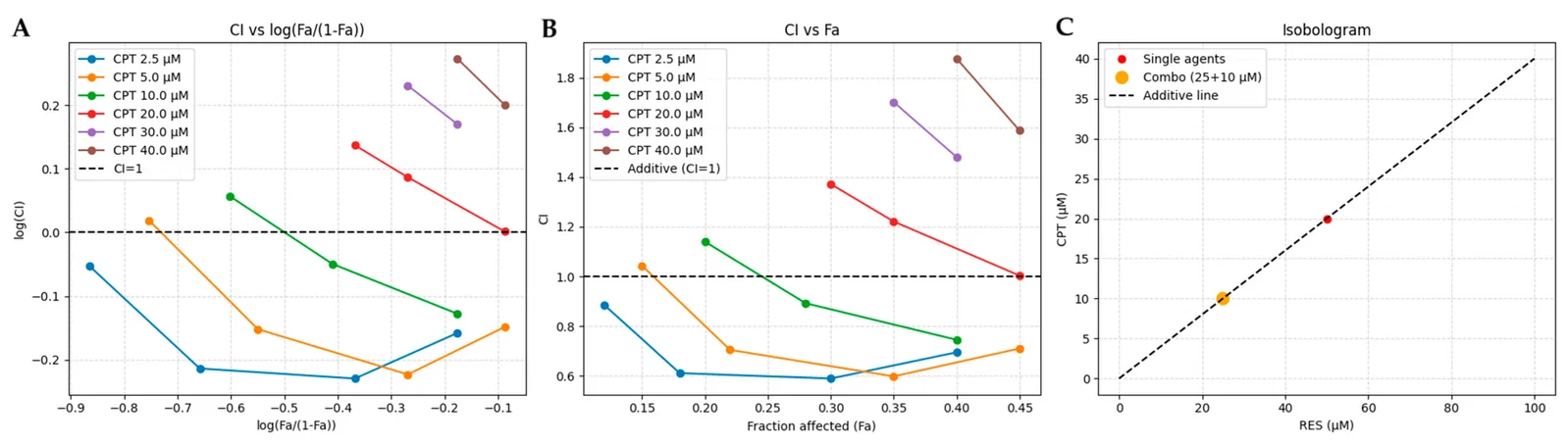
CI and isobologram analysis of the interaction between RES and CPT in Y79 cells. (**A**) CI vs. effect level (Fa) plot. Log(CI) values are plotted against log(Fa/(1 − Fa)). The negative slope (m = −0.18) suggests an increasing trend toward synergism at higher effect levels. All data points fall below CI = 1 (log CI < 0), indicating a potential synergistic interaction across the tested fractions affected. Each data point represents a specific RES and CPT combination dose pair corresponding to a defined fraction affected (Fa). (**B**) CI vs. fraction affected (Fa) plot. CI values at different effect levels (Fa) are shown. The dashed horizontal line represents CI = 1 (additive effect). Data points located below this line (green region) are consistent with synergistic interaction, while values above 1 (red region) represent antagonism. All tested combinations demonstrated CI < 1, suggesting a synergistic trend, particularly at higher Fa values. Each point corresponds to the CI value calculated for an individual combination at a specific Fa level. (**C**) Isobologram analysis. The dashed diagonal line represents the theoretical additive interaction between RES and CPT. The orange point represents the experimental combination treatment, whereas the red points correspond to the single-agent reference doses of RES and CPT used to construct the isobologram. Points below the line denote synergy, whereas points above would indicate antagonism. Overall, CI and isobologram analyses suggest that the combined treatment is associated with a synergistic interaction in Y79 cells.

The interaction of the RES and CPT combination on Y79 cells was evaluated using combination index (CI) analysis with the Chou–Talalay method. The data showed that the CI values were below 1 at all combination points at different effect levels (fraction affected, Fa) (Figure 2A,B). This finding reveals a significant synergistic interaction between RES and CPT.

The log(CI) − log(Fa/(1 − Fa)) graph, obtained by logarithmic transformation of the CI values, showed a negative slope (m = −0.18), indicating that the synergy became more pronounced at increasing effect levels (Figure 2A). The fact that all data points are below the CI = 1 (log CI = 0) line supports the idea that the combination produces an antiproliferative effect beyond the additive effect in all tested dose ranges.

Similarly, in the analysis where CI was plotted directly against Fa, it was determined that all points were positioned below the CI = 1 reference line, and the synergy was stronger, especially at high Fa values (high inhibition levels) (Figure 2B). This indicates that combination therapy is more advantageous at high cytotoxic activity levels.

Isobologram analysis was performed to visually confirm the type of interaction of the combination (Figure 2C). Compared to the diagonal line representing the theoretical additive effect, the experimental combination point (RES 25 µM + CPT 10 µM) was observed to be located below this line. This positioning clearly confirms the synergistic interaction in the relevant dose combination.

**Figure 3:**
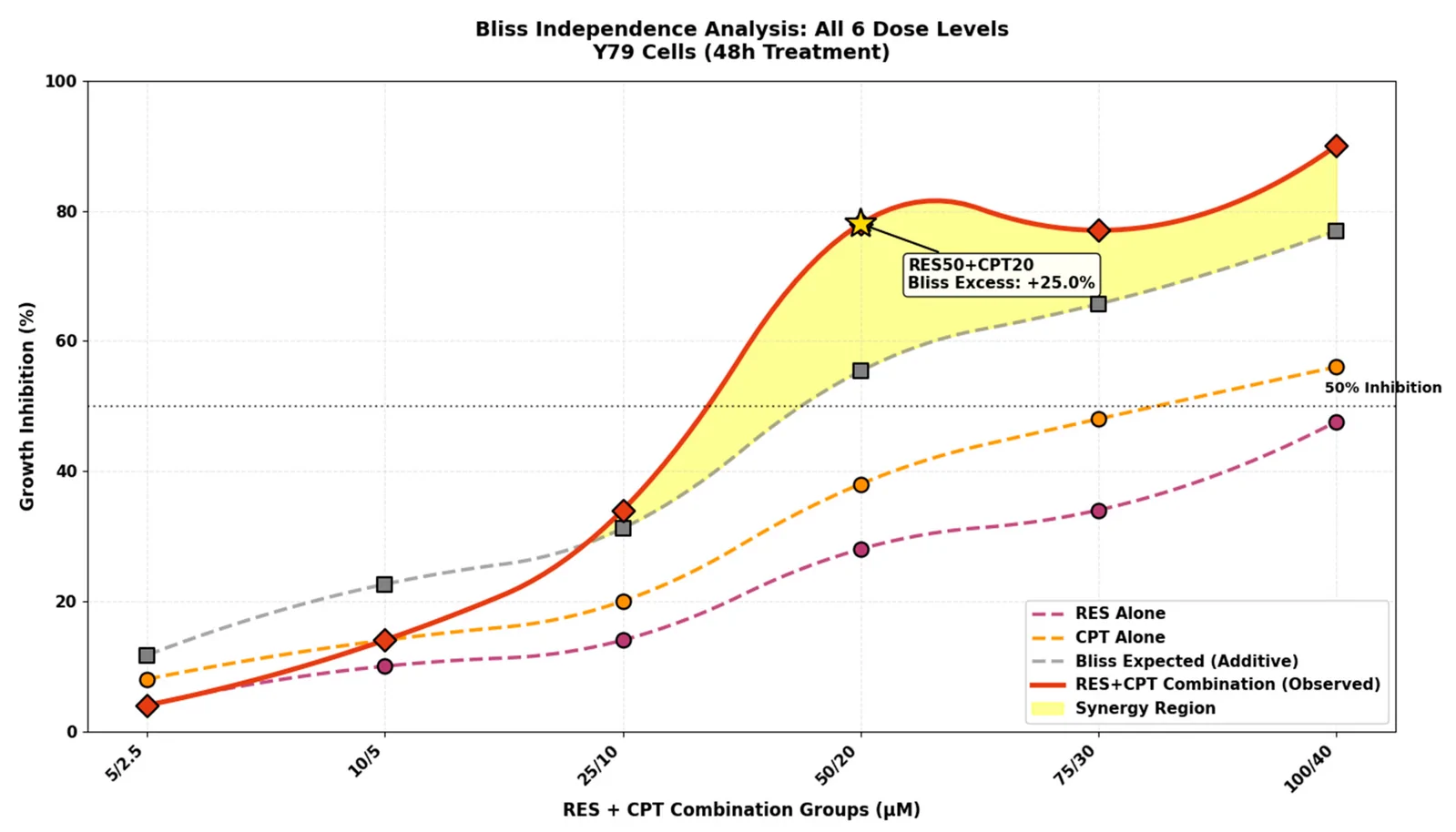
Bliss independence analysis of the interaction between RES and CPT in Y79 cells. Y79 cells were treated with RES (5–100 µM), CPT (2.5–40 µM), or all possible combinations for 48 h. Growth inhibition (%) was calculated relative to the control group. The dashed gray contours represent the expected additive effect according to the Bliss independence model, while the red markers indicate the observed combination effects. The yellow-highlighted points denote regions where the observed inhibition exceeds the Bliss expected value (Bliss excess > 0), indicating potential synergy. For example, the combination of RES50 + CPT20 showed a Bliss excess of +28%, suggesting a strong synergistic interaction at this concentration.

The antiproliferative effect of the RES and CPT combination was evaluated using the Bliss independence model after 48 h of application (Figure 3). While RES and CPT showed gradual inhibition with increasing doses in single-agent applications, the inhibition values obtained in combination applications were found to be higher than the additive effects predicted by Bliss. In low-dose combinations (5/2.5 and 10/5 µM), experimental inhibition values were measured at approximately 4% and 14%, respectively, largely paralleling the expected Bliss values. However, from the medium dose level onwards (25/10 µM), the combination effect increased significantly, and the experimental inhibition (~34%) exceeded the expected Bliss value (~31%). The most significant synergistic effect was observed in the 50 µM RES + 20 µM CPT combination. At this point, experimental inhibition was measured at approximately 78%, while the expected additive inhibition according to the Bliss model was approximately 55%, with a calculated Bliss excess value of +25.0%. This difference clearly demonstrates a strong synergistic interaction.

In higher dose combinations (75/30 and 100/40 µM), experimental inhibition was determined to be approximately 77% and 90%, respectively, and these values were found to be above the expected Bliss inhibition (approximately 66% and 77%). This indicates that the synergistic effect persists over a wide dose range.

**Figure 4:**
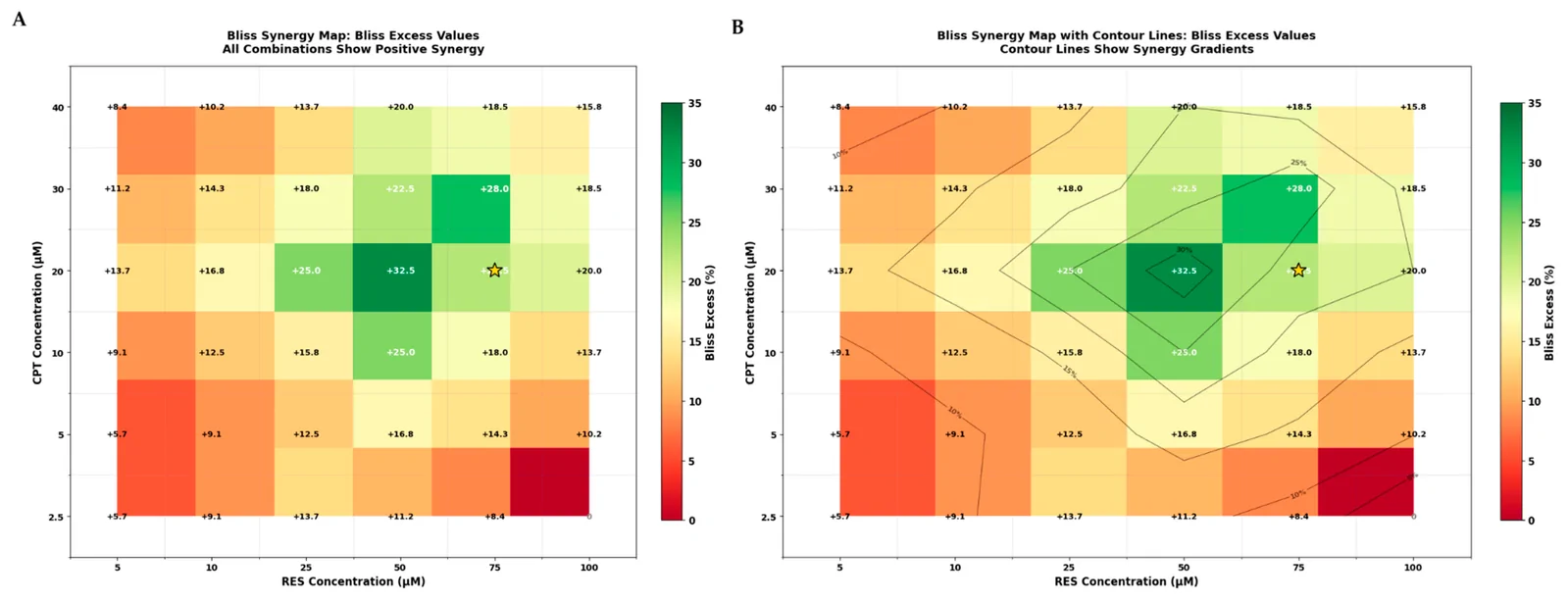
Bliss excess distribution of RES and CPT combinations in Y79 cells. (**A**) Heatmap representing Bliss excess (%) for all tested RES and CPT combinations. Positive Bliss excess values indicate potential synergistic interactions, with darker green colors corresponding to higher synergy. The combination of 50 µM RES and 20 µM CPT exhibited the highest Bliss excess among all tested conditions. (**B**) Contour plot showing the spatial distribution of Bliss excess across the RES × CPT concentration matrix. Contour lines delineate increasing levels of Bliss excess, highlighting that maximal synergy occurs at intermediate concentrations, such as RES 50 µM and CPT 20 µM.

Graphical analysis clearly shows that the combination curve lies above the expected Bliss curve across all dose ranges, and the area between the two curves (synergy region) is positive. Furthermore, it is noteworthy that the 50% inhibition level is exceeded at lower effective doses in combination therapy (Figure 3 and Figure 4).

### 2.3. RES-CPT Combination Induces Apoptosis and G2/M Cell Cycle Arrest in Y79 Cells

**Figure 5:**
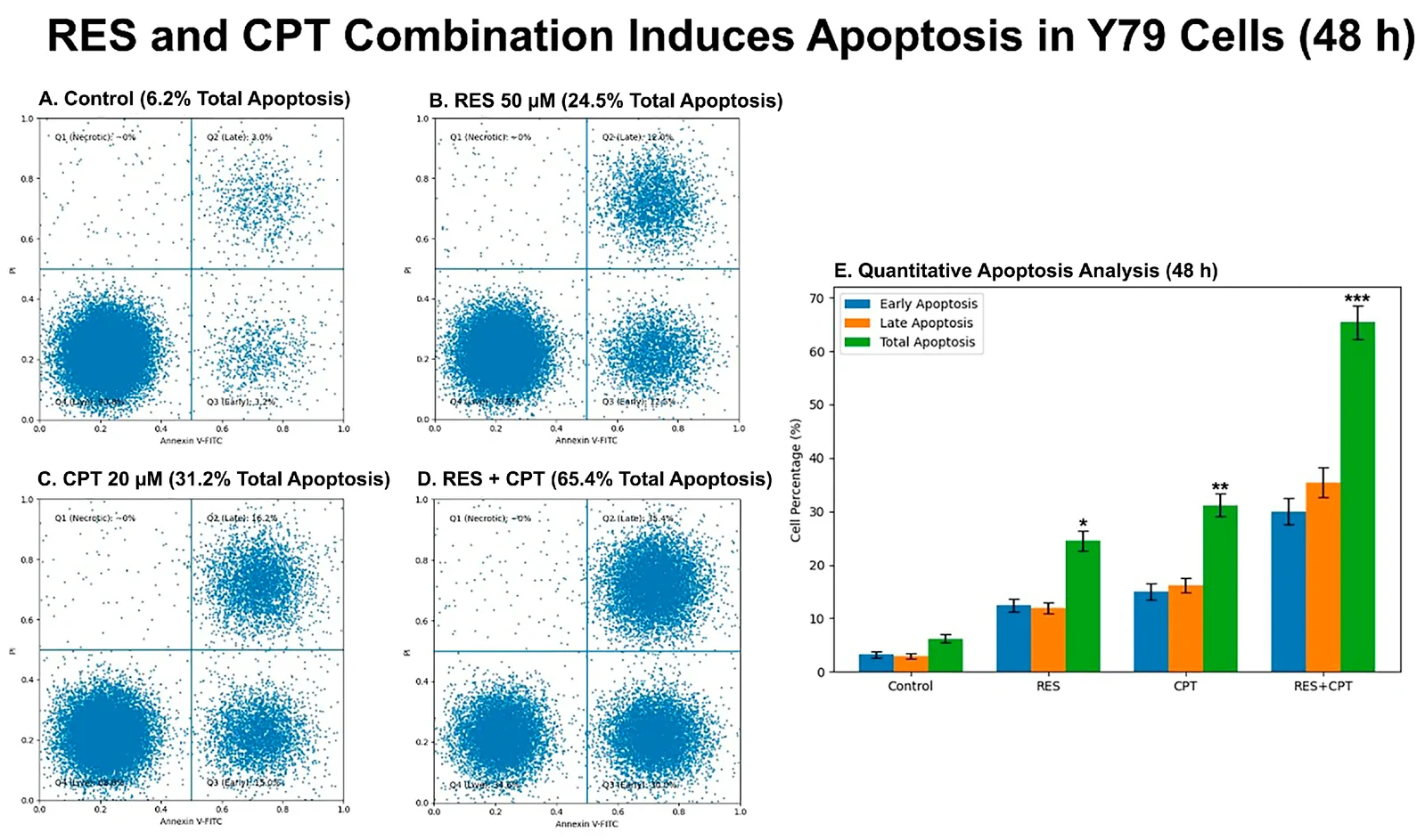
Annexin V-FITC/PI-based assessment of apoptosis in Y79 cells following 48 h treatment with RES and CPT. (**A**–**D**) Representative flow cytometry dot plots showing the distribution of viable (Q4), early apoptotic (Q3), late apoptotic (Q2), and necrotic (Q1) cell populations after treatment with control (0.1% DMSO), RES (50 µM), CPT (20 µM), or RES + CPT combination. Quadrant percentages indicate the proportion of cells within each gated region. Total apoptosis was calculated as the sum of early and late apoptotic populations (Q3 + Q2). (**E**) Quantitative analysis of early, late, and total apoptotic cell percentages. Data are presented as mean ± SD from three independent experiments. Statistical comparisons were performed relative to the control group using one-way ANOVA followed by Tukey’s multiple comparison test (* *p* < 0.05, ** *p* < 0.01, *** *p* < 0.001).

The apoptotic effects of the drugs were analyzed by flow cytometry with Annexin V-FITC/PI staining. After 48 h of treatment, the total apoptotic cell (early + late apoptosis) ratio was 6.2 ± 1.1% in the control group, while 50 µM RES alone increased this ratio to 24.5 ± 2.8%, and 20 µM CPT alone increased it to 31.2 ± 3.1%. The combination of 50 µM RES and 20 µM CPT increased the total apoptotic cell ratio to 65.4 ± 4.3% (*p* < 0.001, combination vs. single agents). Representative flow cytometry dot plots are shown in Figure 5A, and quantitative analysis of apoptotic populations is presented in Figure 5B.

In control cells, the G2/M phase population was 38.5 ± 2.1%, whereas treatment with the RES + CPT combination significantly increased this proportion to 71.8 ± 3.2% (*p* < 0.001). This increase was accompanied by a corresponding reduction in the G0/G1 population, indicating a marked blockade at the G2/M checkpoint. These findings demonstrate that the RES + CPT combination induces cell cycle arrest at the G2/M checkpoint.

**Figure 6:**
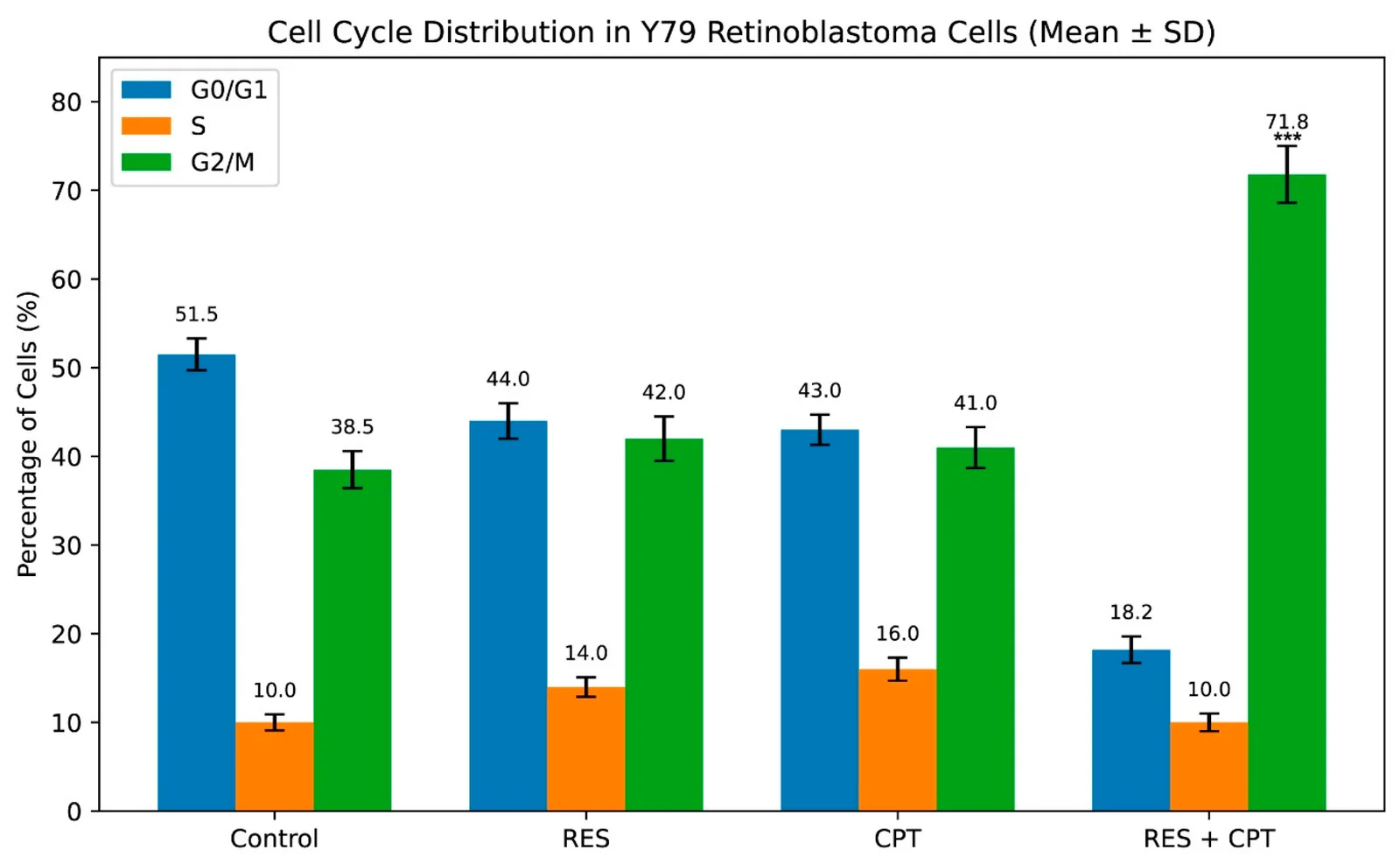
Effects of RES and CPT on cell cycle distribution in Y79 RB cells. Y79 cells were treated with RES (50 µM), CPT (20 µM), or their combination (RES + CPT) for 48 h. The percentage of cells in G0/G1, S, and G2/M phases was determined by flow cytometric analysis of DNA content. Data are presented as mean ± SD (n = 3). The RES + CPT combination was associated with an increase in the G2/M population compared to the control group (*** *p* < 0.001). A decrease in the G0/G1 fraction was also observed in the combination group. Statistical analysis was performed using one-way ANOVA followed by Tukey’s post hoc test.

Cell cycle analysis revealed a marked alteration in phase distribution following combination treatment. While RES and CPT monotherapies induced modest changes in cell cycle progression, the RES + CPT group demonstrated a pronounced accumulation of cells in the G2/M phase (71.8 ± 3.2%) compared with the control (38.5 ± 2.1%), RES (42.0 ± 2.5%), and CPT (41.0 ± 2.3%) groups (*** *p* < 0.001). This increase was accompanied by a corresponding reduction in the G0/G1 population, indicating effective blockade at the G2/M checkpoint (Figure 6).

### 2.4. RES–CPT Co-Treatment Promotes Mitochondrial Depolarization and ROS Accumulation in Y79 Cells

**Figure 7:**
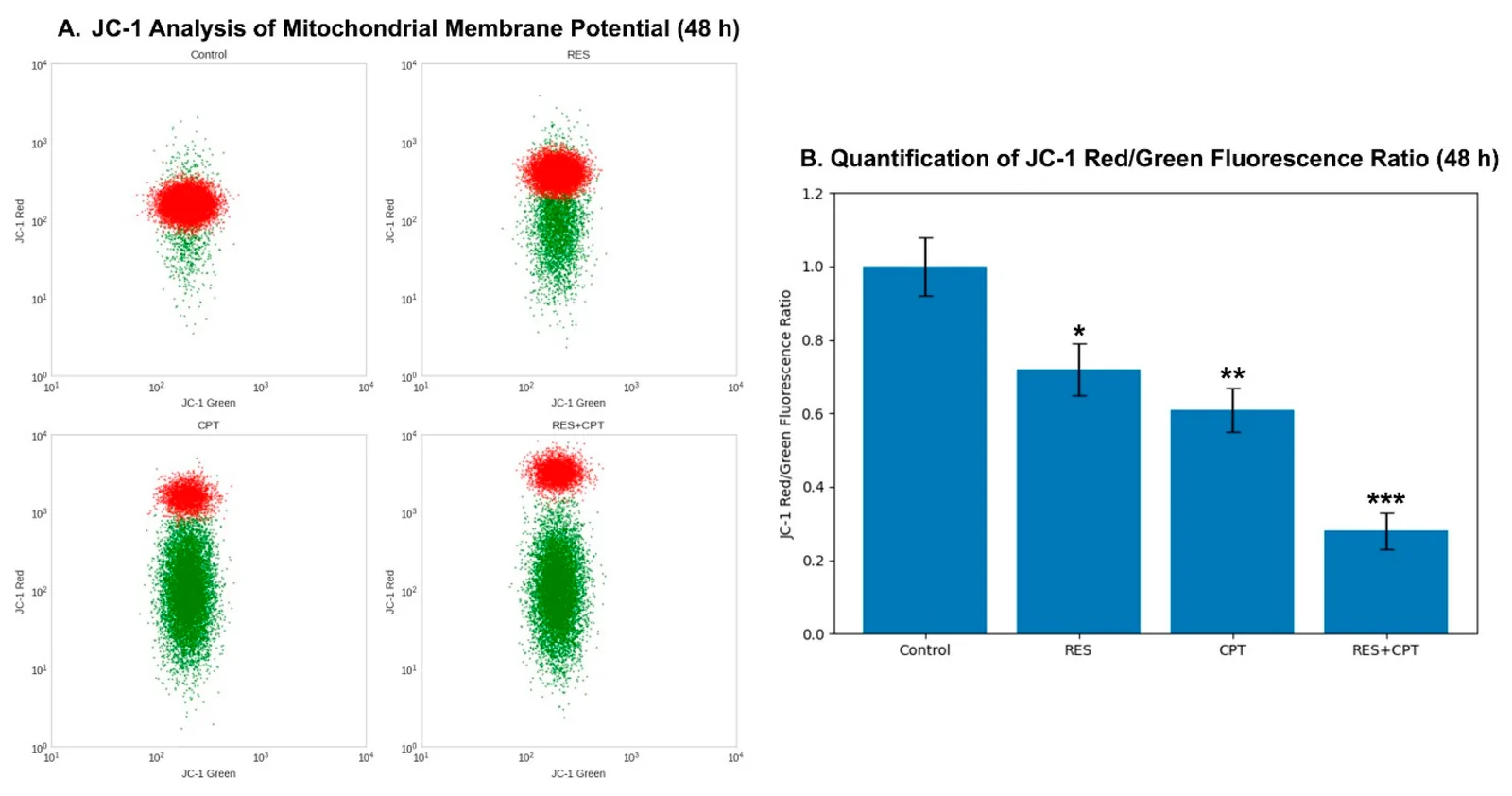
JC-1-based assessment of mitochondrial membrane potential in Y79 cells following 48 h treatment with RES and CPT. (**A**) Representative JC-1 flow cytometry dot plots illustrating mitochondrial membrane potential after treatment with control (0.1% DMSO), RES (50 µM), CPT (20 µM), or RES + CPT combination. Red fluorescence (JC-1 aggregates) indicates intact mitochondrial membrane potential, whereas green fluorescence (JC-1 monomers) reflects mitochondrial depolarization. (**B**) Quantitative analysis of the JC-1 red/green fluorescence ratio. Data are presented as mean ± SD from three independent experiments. Statistical comparisons were performed relative to the control group using one-way ANOVA followed by Tukey’s multiple comparison test (* *p* < 0.05, ** *p* < 0.01, *** *p* < 0.001).

The effects of combination therapy on mitochondrial function were evaluated using JC-1 staining. The red/green fluorescence ratio was reduced by approximately 72% in the combination group compared with the control (*p* < 0.001), indicating marked mitochondrial membrane depolarization. Although partial depolarization was observed following single treatments, the RES + CPT combination induced the most pronounced loss of mitochondrial membrane potential (Figure 7A,B). These findings are consistent with activation of the intrinsic (mitochondrial) apoptotic pathway.

### 2.5. RES–CPT Combination Significantly Enhances Intracellular ROS Production in Y79 Cells

**Figure 8:**
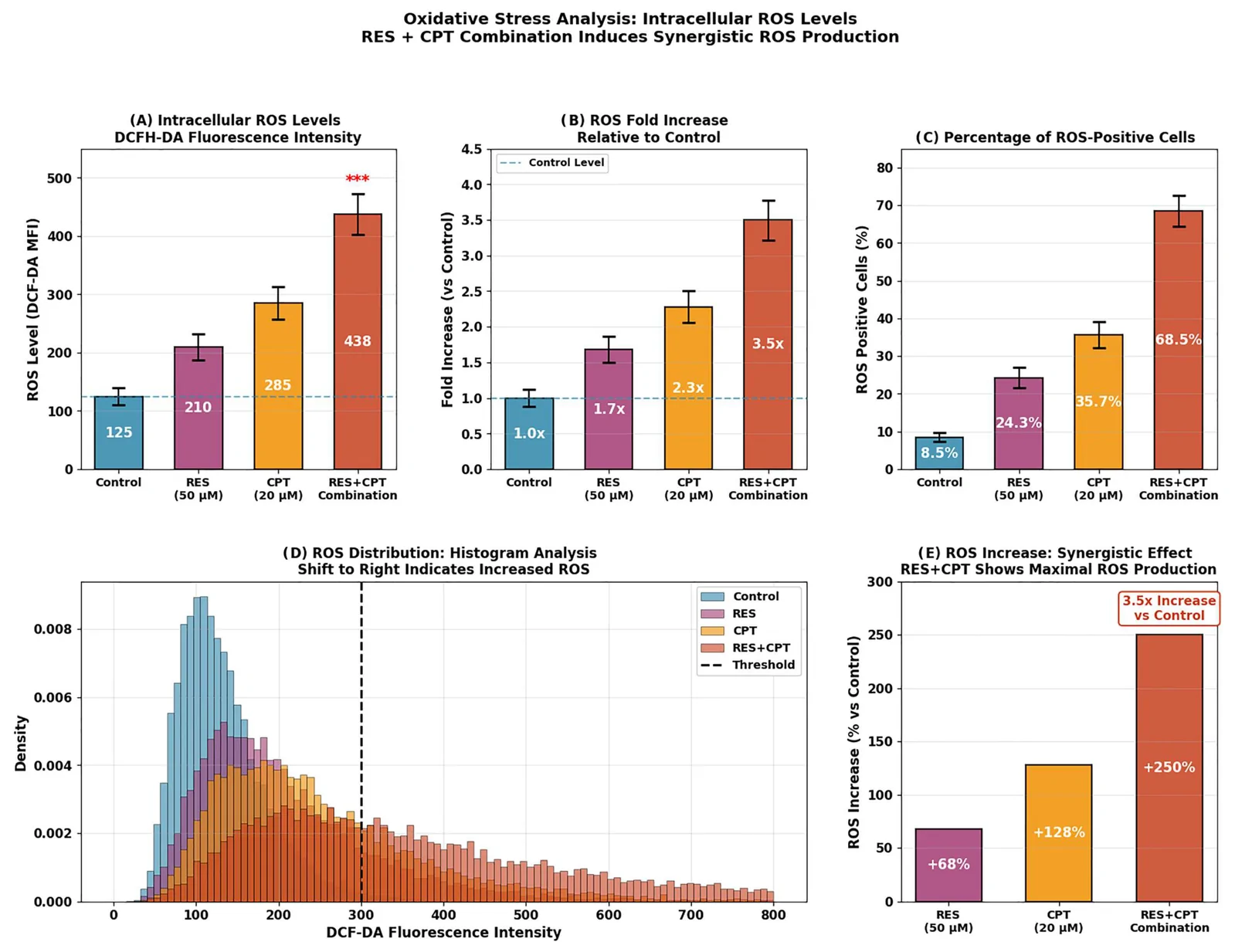
Assessment of intracellular ROS levels in Y79 cells following RES and CPT treatment. (**A**) Mean fluorescence intensity (MFI) of DCFH-DA reflecting intracellular ROS levels. Compared to the control group, both RES and CPT monotherapies significantly increased ROS levels, while the RES + CPT combination showed the highest increase (*** *p* < 0.001). The dashed line represents the control baseline. (**B**) Fold increase in ROS levels relative to control. (**C**) Percentage of ROS-positive cells in each treatment group. (**D**) Representative histogram overlay of DCFH-DA fluorescence intensity. A rightward shift in fluorescence distribution is observed in treated groups, particularly in the RES + CPT group, consistent with increased ROS levels. The dashed vertical line denotes the positivity threshold. (**E**) Comparative analysis of the increase in ROS. The observed ROS levels in the combination group were higher than those of individual treatments and the calculated additive reference. The expected additive effect was calculated using the Bliss independence model and represents the theoretical combined effect of RES and CPT, not NAC treatment. This pattern suggests a potential cooperative effect of the combination on ROS production. Data are presented as mean ± SD from three independent experiments. Statistical comparisons were performed relative to the control group using one-way ANOVA followed by Tukey’s multiple comparison test.

Intracellular reactive oxygen species (ROS) levels were assessed using the DCFH-DA fluorescence probe to evaluate oxidative stress induced by RES, CPT, and their combination. As shown in Figure 8A, treatment with RES (50 µM) or CPT (20 µM) alone resulted in moderate increases in ROS levels compared to the control group. Notably, the combination of RES and CPT led to a marked and statistically significant elevation in ROS production, with mean fluorescence intensity (MFI) reaching 438 ± 35, approximately 3.5-fold higher than control (*p* < 0.001) and significantly higher than either monotherapy (*p* < 0.01). The percentage of ROS-positive cells was also highest in the combination group (68.5 ± 4.2%) (Figure 8C). Furthermore, histogram analysis revealed a pronounced rightward shift in fluorescence intensity in combination-treated cells, confirming robust ROS accumulation (Figure 8D). These findings indicate that the RES + CPT combination synergistically amplifies oxidative stress in Y79 cells.

### 2.6. NAC Pretreatment Attenuates ROS Accumulation and Partially Restores Cell Viability in Y79 Cells

To investigate whether the observed increase in intracellular ROS levels is causally involved in the synergistic cytotoxic effect of the RES + CPT combination, cells were pretreated with N-acetylcysteine (NAC), a well-established ROS scavenger. NAC functions as a precursor of glutathione (GSH) synthesis and directly neutralizes free radicals, thereby reducing oxidative stress. By depleting ROS, NAC enables the assessment of whether ROS accumulation is a critical mediator of the observed cell death. This approach allows us to mechanistically validate the functional contribution of oxidative stress to the antiproliferative effects of the combination therapy.

**Figure 9:**
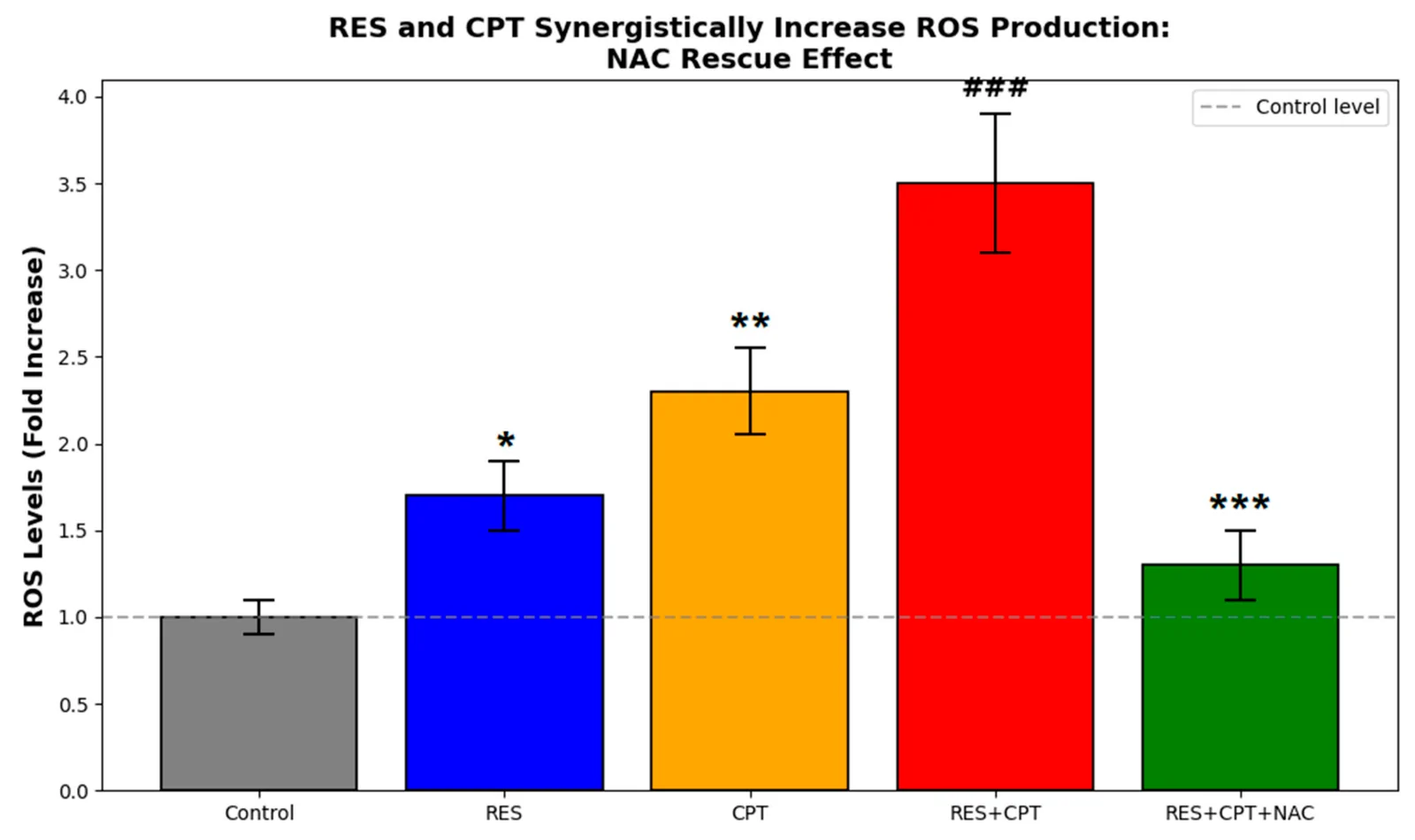
Effects of RES, CPT, and their combination with or without NAC on intracellular ROS levels in Y79 RB cells. Cells were treated with RES (50 µM), CPT (20 µM), RES + CPT combination, or RES + CPT combined with NAC (5 mM) for 48 h. Intracellular ROS levels were expressed as fold change relative to the control group. Data are presented as mean ± SD from three independent experiments (n = 3). Statistical analysis was performed using one-way ANOVA followed by Tukey’s post hoc test. * *p* < 0.05, ** *p* < 0.01, *** *p* < 0.001 vs. control; ^###^ *p* < 0.001 vs. RES + CPT group. NAC treatment reduced ROS levels in RES + CPT-treated cells, indicating an attenuation of ROS accumulation under these conditions.

Whether ROS production plays a causal role in the mechanism of action of the RES + CPT combination was investigated using a rescue assay with the antioxidant NAC. When cells were co-treated with the RES + CPT combination and NAC (5 mM), the ROS levels were significantly reduced to 1.3-fold compared to the control (*p* < 0.001 vs. RES + CPT combination) (Figure 9).

Pretreatment with NAC (5 mM) markedly attenuated ROS accumulation in RES + CPT-treated cells, as evidenced by a significant reduction in DCF fluorescence intensity. NAC alone did not significantly alter intracellular ROS levels relative to control.

Flow cytometry histograms confirmed that RES + CPT treatment induced a clear rightward shift in DCF fluorescence intensity, which was substantially reduced following NAC pretreatment.

**Figure 10:**
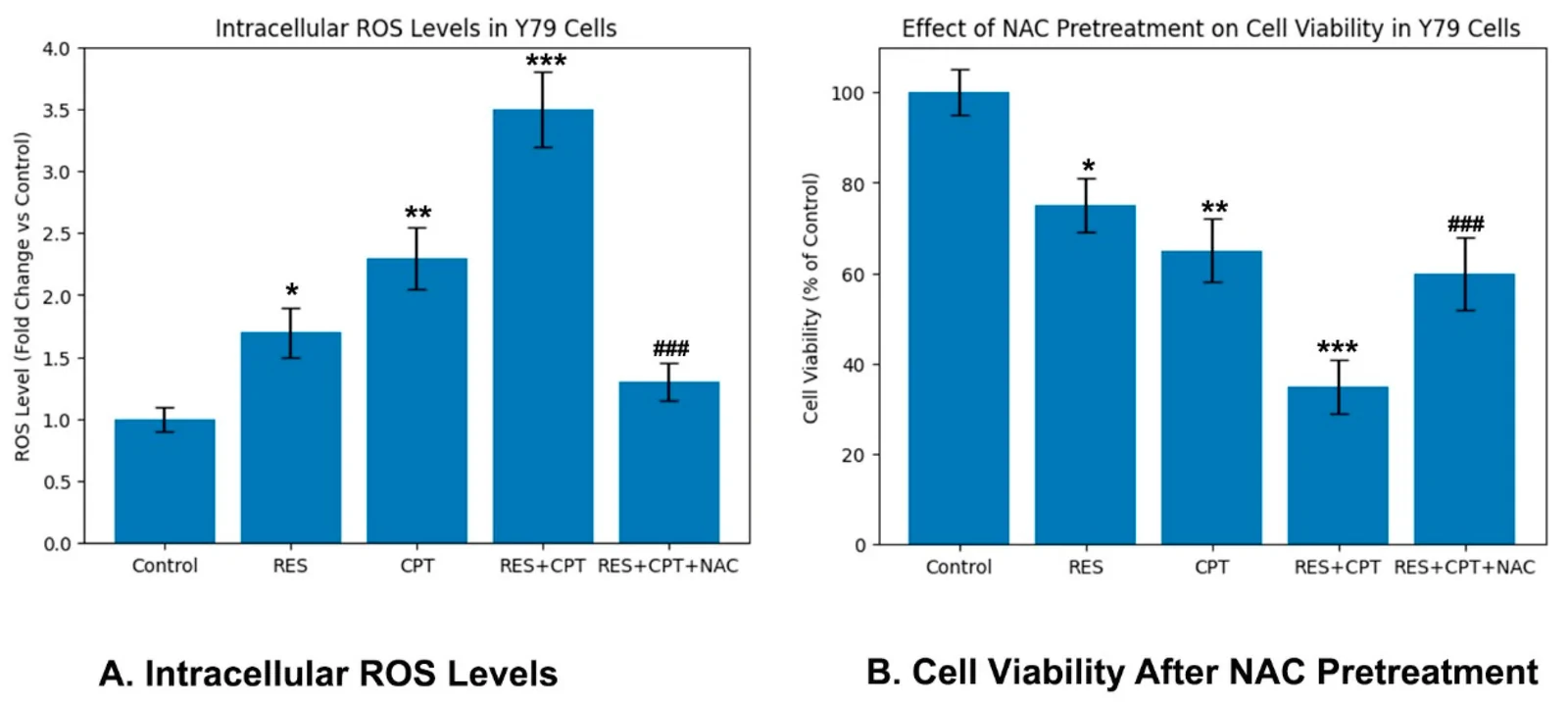
Effect of NAC on intracellular ROS levels and cell viability in Y79 RB cells. (**A**) Intracellular ROS levels measured by DCFH-DA assay and expressed as fold change relative to control. (**B**) Cell viability assessed by MTT assay and expressed as a percentage of the control. Data represent mean ± SD (n = 3). * *p* < 0.05, ** *p* < 0.01, *** *p* < 0.001 vs. control; ^###^ *p* < 0.001 vs. the RES + CPT combination. NAC treatment reduced ROS levels and partially restored cell viability in RES + CPT-treated cells.

Furthermore, NAC pretreatment partially restored cell viability in RES + CPT-treated cells, although viability did not fully return to control levels. These findings indicate that excessive ROS generation plays a mechanistic role in the enhanced cytotoxic effect observed with RES and CPT co-treatment (Figure 10).

**Figure 11:**
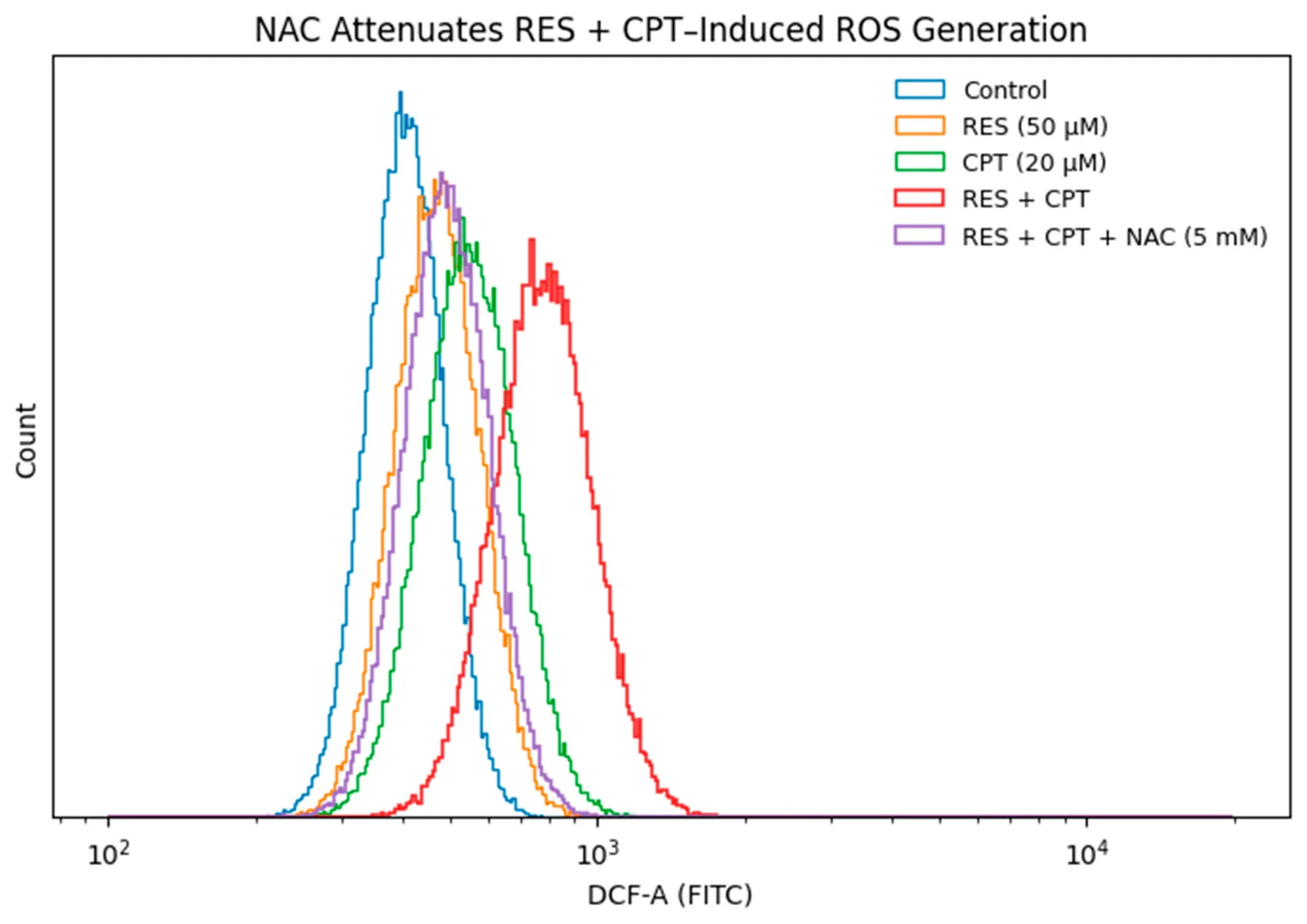
Flow cytometric histogram representation of intracellular ROS levels in Y79 cells. Representative overlay histograms of DCF fluorescence intensity (DCFH-DA assay) in Y79 cells treated with RES (50 µM), CPT (20 µM), RES + CPT, and RES + CPT + NAC (5 mM) for 48 h. The RES + CPT combination shows a rightward shift in fluorescence intensity compared with the control and monotherapy groups, consistent with increased ROS levels. NAC pretreatment partially reduces this shift, indicating attenuation of ROS levels under these conditions.

Consistent with these findings, representative overlay flow cytometry histograms demonstrated a pronounced rightward shift in DCF fluorescence intensity following RES + CPT treatment, which was substantially attenuated by NAC pretreatment, confirming ROS modulation at the single-cell level (Figure 11).

### 2.7. RES-CPT Combination Enhances Caspase-3 Enzymatic Activity in Y79 Cells

**Figure 12:**
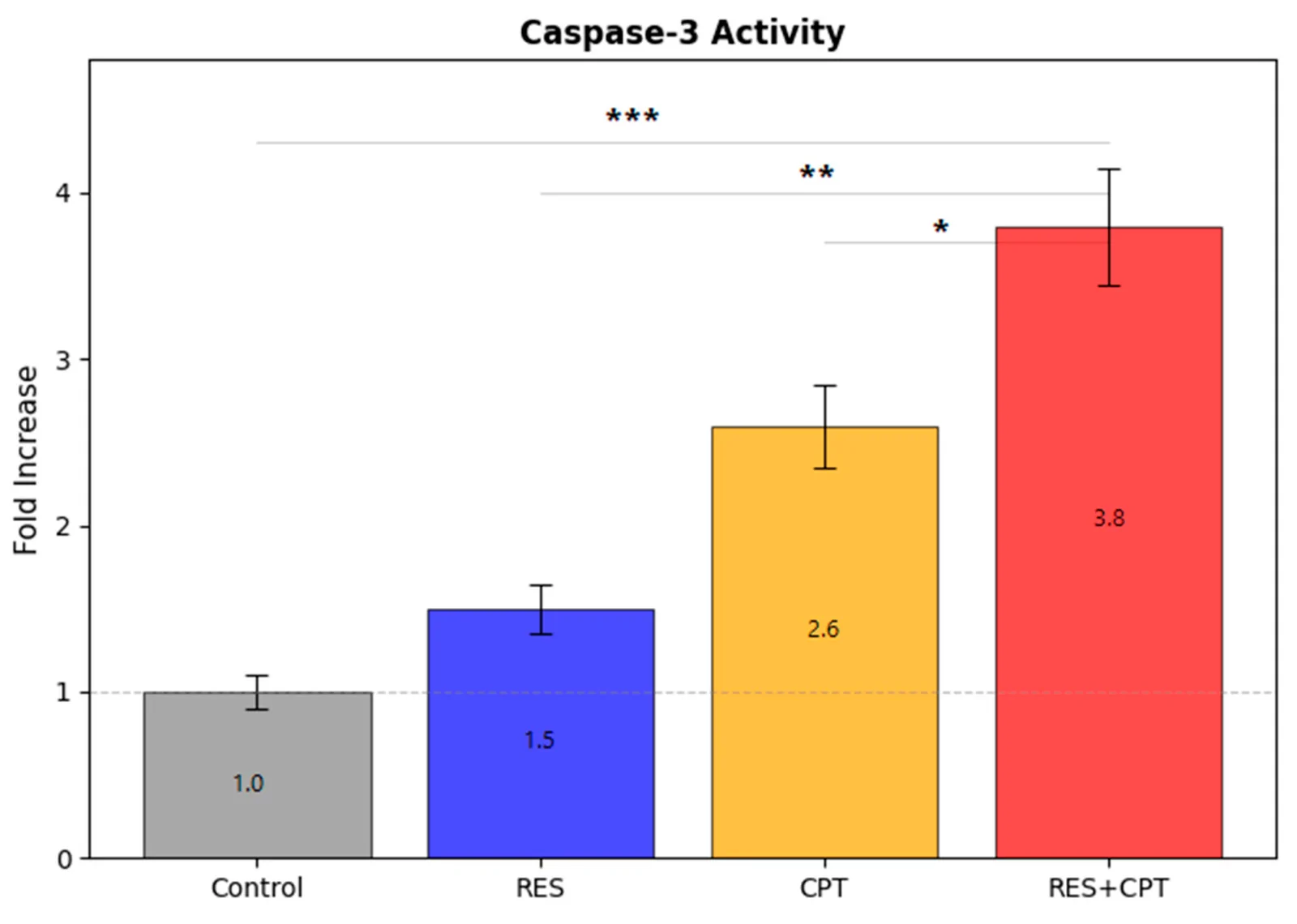
Effects of RES, CPT, and their combination on caspase-3 activity in Y79 RB cells. Caspase-3 activity was measured colorimetrically using a specific chromogenic substrate and expressed as fold change relative to the control group. Data are presented as mean ± SD of three independent experiments. Statistical analysis was performed using one-way ANOVA followed by Tukey’s multiple comparison test. Statistical significance is indicated as * *p* < 0.05, ** *p* < 0.01, *** *p* < 0.001 for comparisons between groups as shown in the figure.

In the analysis of caspase-3 enzyme activity, RES alone resulted in a 1.5-fold increase and CPT a 2.6-fold increase. The combination treatment increased activity 3.8-fold. This increase is significantly higher than that of single treatments and indicates functional activation of the intrinsic apoptotic pathway (Figure 12).

### 2.8. RES-CPT Co-Treatment Modulates Apoptosis and Cell Cycle-Related Gene Expression in Y79 Cells

**Figure 13:**
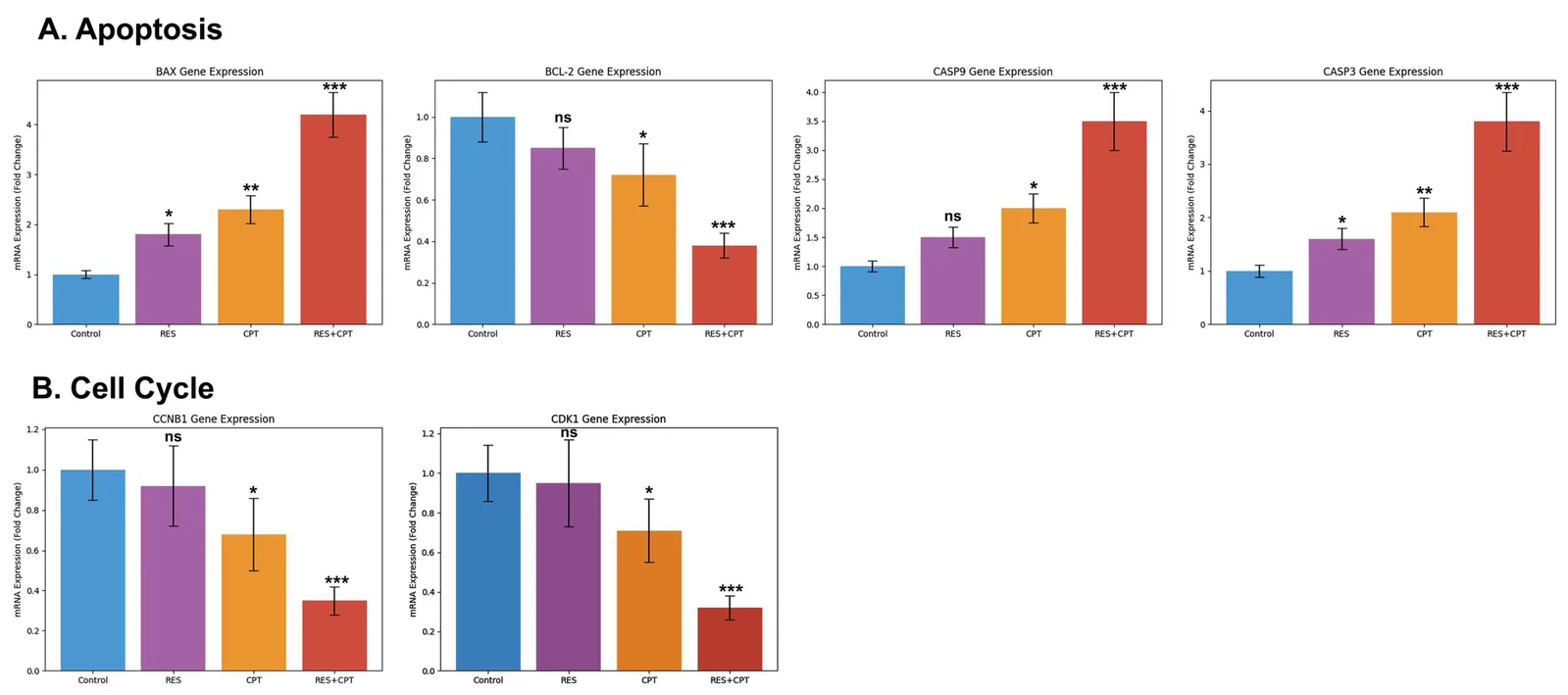
Effects of RES and CPT, alone and in combination, on apoptosis- and cell cycle-related gene expression in Y79 cells. (**A**) Relative mRNA expression levels of apoptosis-related genes (*BAX*, *BCL-2*, *CASP9*, and *CASP3*). (**B**) Relative mRNA expression levels of cell cycle-related genes (CCNB1 and CDK1). Relative gene expression levels were determined by qRT-PCR following treatment with RES (IC_50_), CPT (IC_50_), and their combination for 48 h. Treatment with RES and CPT individually was associated with increased expression of pro-apoptotic genes (*BAX*, *CASP9*, and *CASP3*), whereas the combined treatment resulted in more pronounced upregulation. In contrast, *BCL-2* expression showed a decreasing trend in single-treatment groups and was markedly reduced in the RES + CPT combination group. Regarding cell cycle-related genes, *CCNB1* and *CDK1* expression levels exhibited modest reductions following monotherapies and were further decreased in the combination group, consistent with enhanced suppression of cell cycle progression. Data are presented as mean ± SD of three independent experiments. Statistical significance is indicated as * *p* < 0.05, ** *p* < 0.01, and *** *p* < 0.001 versus control; ns: not significant.

RT-qPCR analyses showed that combination therapy produced significant changes at the transcriptional level. Y79 cells were treated with RES (50 µM), CPT (20 µM), and their combination for 48 h, and mRNA expression levels of *BAX*, *BCL-2*, *CASP9*, *CASP3*, *CCNB1* (cyclin B1), and *CDK1* were analyzed. The results are summarized in Figure 13. The combination therapy significantly upregulated the mRNA expression of the pro-apoptotic gene *BAX* (4.2-fold increase vs. control; *p* < 0.001) and the effector caspase *CASP3* (3.8-fold increase; *p* < 0.001). Similarly, the initiator caspase *CASP9* showed a 3.5-fold increase in expression (*p* < 0.01). In contrast, the anti-apoptotic gene BCL-2 was significantly downregulated by the combination treatment (0.38-fold change vs. control; *p* < 0.01). The *BAX*/*BCL-2* mRNA ratio, a key determinant of mitochondrial apoptotic pathway activation, was dramatically increased in the combination group compared to control, RES alone, and CPT alone (*p* < 0.001 for all comparisons). This sharp increase in the *BAX*/*BCL-2* ratio strongly indicates the activation of the intrinsic apoptotic pathway at the transcriptional level. The expression of the cell cycle regulators *CCNB1* and *CDK1* genes was decreased to 0.35-fold and 0.32-fold, respectively. These results are consistent with the observed G2/M phase blockade (Figure 13).

### 2.9. RES-CPT Co-Treatment Induces Cytoskeletal Disruption and Apoptotic Morphology in Y79 Cells

**Figure 14:**
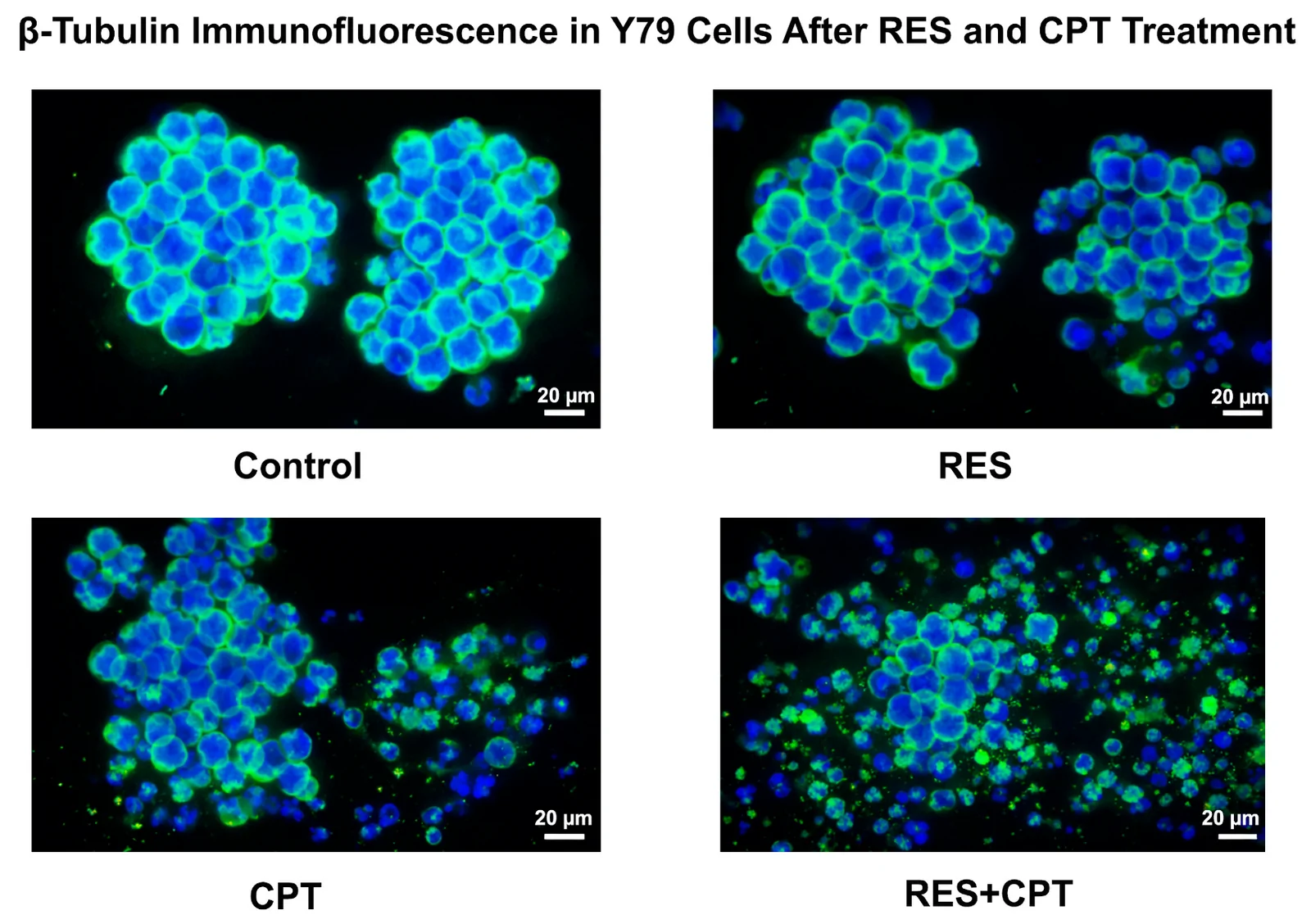
Immunocytochemical assessment of β-tubulin organization in Y79 cells following treatment with RES and CPT. Representative fluorescence microscopy images of Y79 RB cells subjected to Control, RES (50 µM), CPT (20 µM), and RES + CPT combination treatments for 48 h. Cells were immunostained with an anti-β-tubulin antibody (green) to visualize microtubule structures and counterstained with DAPI (blue) to label nuclei. In the control group, cells exhibited compact cluster formation with relatively uniform microtubule distribution. RES and CPT-treated groups showed alterations in microtubule organization and changes in nuclear morphology. In contrast, the RES + CPT combination group displayed more pronounced disruption of cellular organization and increased nuclear condensation, suggestive of enhanced cellular stress and apoptotic features. Scale bar: 20 µm.

Immunocytochemical analysis of β-tubulin was performed to evaluate cytoskeletal integrity and microtubule organization in Y79 cells following treatment with RES IC_50_, CPT IC_50_, and their combination IC_50_ for 48 h. In the control group, β-tubulin staining revealed a well-organized and uniformly distributed microtubule network. Cells maintained compact cluster formation with intact cytoskeletal architecture and regular, round nuclear morphology as confirmed by DAPI counterstaining. In the RES monotherapy group, mild alterations in microtubule organization were observed. Although the overall cytoskeletal structure was largely preserved, partial disruption and slight disorganization of β-tubulin filaments were evident. Nuclear morphology showed limited condensation, consistent with moderate cellular stress. In the CPT-treated group, more pronounced cytoskeletal disruption was detected. β-tubulin staining intensity appeared reduced in certain regions, and microtubule organization was less uniform. Cells displayed morphological features associated with apoptosis, including cytoplasmic shrinkage and nuclear condensation. The RES + CPT combination group demonstrated the most substantial alterations in β-tubulin organization. Marked disassembly and fragmentation of the microtubule network were observed, accompanied by decreased structural integrity and irregular cytoskeletal distribution. Additionally, prominent nuclear condensation and increased cellular fragmentation were evident, consistent with advanced apoptotic morphology. Collectively, these findings indicate that while RES and CPT individually induce moderate cytoskeletal alterations, their combination markedly disrupts β-tubulin organization, suggesting enhanced cytoskeletal destabilization associated with synergistic apoptotic induction in Y79 cells (Figure 14).

### 2.10. Evaluation of the Effects of RES and CPT in a 3D Tumor Spheroid Model

#### 2.10.1. Formation and Morphological Alterations of 3D Tumor Spheroids

To further investigate the antitumor effects of RES and CPT under conditions that better mimic tumor architecture, Y79 RB cells were cultured as three-dimensional (3D) tumor spheroids using ultra-low attachment plates. As described in the Section 4, treatment conditions were applied following spheroid formation.

Under control conditions, Y79 cells formed compact, spherical, and well-defined spheroids with smooth and regular borders. RES treatment (50 µM) resulted in a moderate reduction in spheroid size while largely preserving spheroid integrity and overall architecture. In contrast, CPT treatment (20 µM) induced a more pronounced decrease in spheroid diameter accompanied by partial disruption of compactness.

**Figure 15:**
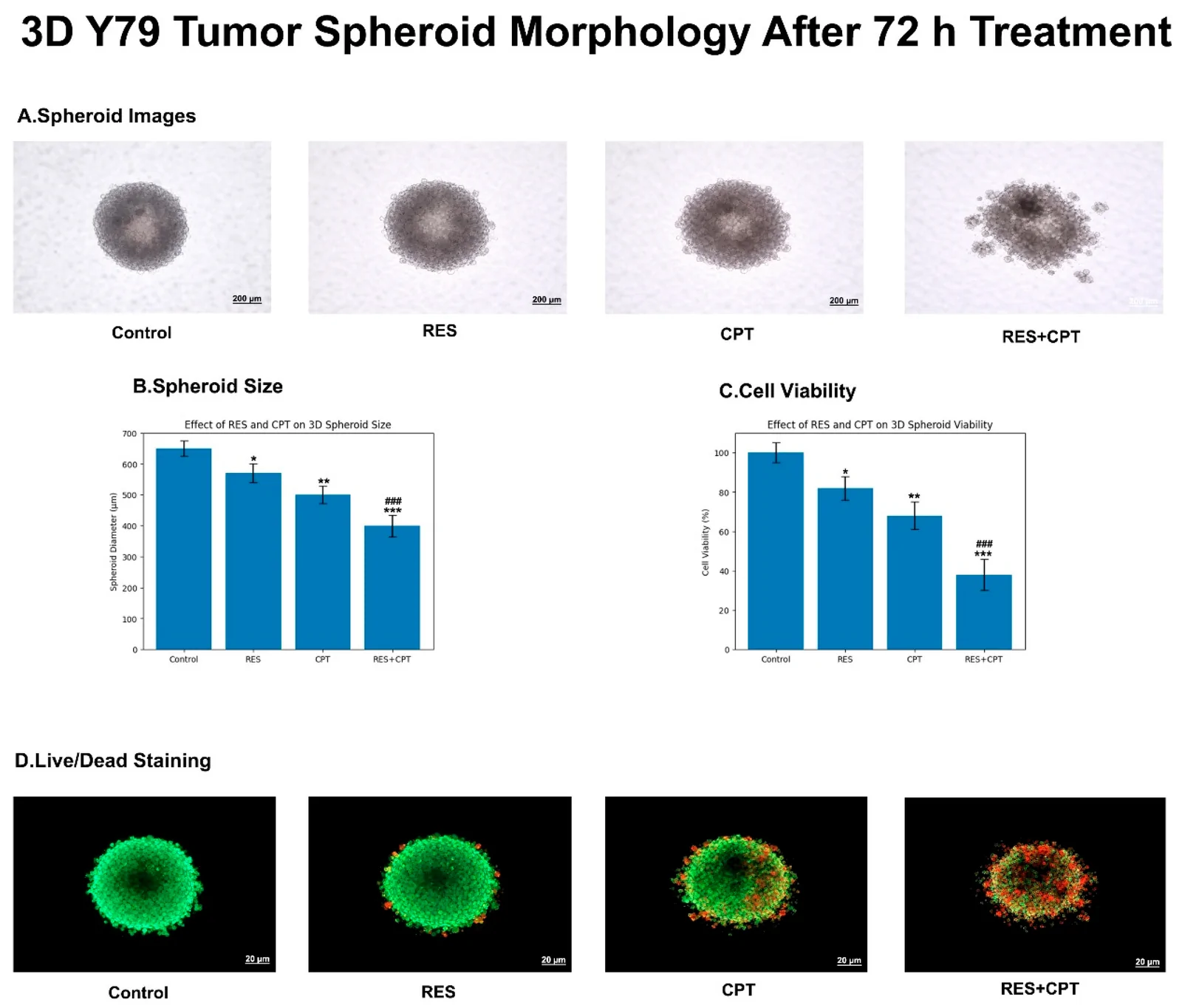
Bright-field images and live/dead fluorescence imaging of 3D Y79 tumor spheroids following 72 h treatment with RES and CPT. (**A**) Bright-field images of control, RES-, CPT-, and RES + CPT-treated spheroids (scale bar: 200 µm). (**B**) Quantitative analysis of spheroid diameter. (**C**) ATP-based cell viability assessment. (**D**) Live/Dead fluorescence images showing viable (green) and dead (red) cells (scale bar: 20 µm). Data are presented as mean ± SD (n = 3). Statistical analysis was performed using one-way ANOVA followed by Tukey’s multiple comparison test. Statistical significance is indicated as * *p* < 0.05, ** *p* < 0.01, *** *p* < 0.001 vs. control, and ^###^ *p* < 0.001 for comparisons as indicated in the figure.

Notably, combined RES and CPT treatment led to marked spheroid shrinkage and structural disorganization. Spheroids in the combination group exhibited irregular borders, reduced compactness, and fragmented peripheral regions, indicating enhanced structural impairment compared with single-agent treatments (Figure 15A).

#### 2.10.2. Quantitative Reduction in Spheroid Size Following RES and CPT Co-Treatment

Quantitative analysis of spheroid diameter demonstrated significant differences among experimental groups. Compared with control spheroids, RES treatment significantly reduced spheroid diameter (*p* < 0.05), whereas CPT treatment exerted a greater inhibitory effect (*p* < 0.01 vs. control).

The most pronounced reduction in spheroid size was observed in the RES + CPT group, in which the mean spheroid diameter was significantly lower than that observed in both the RES and CPT monotherapy groups (*p* < 0.001). These findings indicate that co-treatment with RES and CPT potentiates growth inhibition in the 3D tumor spheroid model (Figure 15B).

#### 2.10.3. Suppression of Cell Viability in 3D Tumor Spheroids by RES and CPT Combination

Cell viability within 3D tumor spheroids was assessed using an ATP-based CellTiter-Glo^®^ 3D assay. RES treatment alone produced a modest but statistically significant reduction in spheroid viability compared with control (*p* < 0.05). CPT treatment induced a more substantial decrease in metabolic activity (*p* < 0.01).

Importantly, combined RES and CPT treatment resulted in the strongest cytotoxic effect, significantly reducing spheroid viability compared with both control and single-agent treatments (*p* < 0.001). These results indicate that the synergistic cytotoxic interaction observed in 2D cultures is maintained in the 3D tumor spheroid model (Figure 15C).

#### 2.10.4. Live/Dead Fluorescence Staining Reveals Enhanced Cell Death in RES + CPT-Treated Spheroids

To visualize treatment-induced cytotoxicity, live/dead fluorescence staining was performed on 3D tumor spheroids. Control spheroids predominantly exhibited green fluorescence, indicating a high proportion of viable cells and preserved structural integrity.

RES-treated spheroids showed increased red fluorescence compared with the control, reflecting moderate cell death. CPT-treated spheroids demonstrated a more evident increase in red fluorescence, accompanied by partial disruption of spheroid organization.

In contrast, spheroids treated with the RES + CPT combination displayed extensive red fluorescence throughout the spheroid structure, together with a marked reduction in green fluorescence and disruption of spheroid architecture. These findings support the enhanced cytotoxic and apoptotic effects of the combination treatment in a 3D tumor context (Figure 15D).

### 2.11. In Silico Target Prediction and Network Interaction Analysis of RES and CPT

To support experimental findings and predict potential target proteins, a comprehensive in silico analysis was performed for RES and CPT. Target screening was performed using SwissTargetPrediction and PharmMapper servers, based on the chemical structure of RES (PubChem) and the platinum nitrogen coordination structure of CPT.

RES-Specific Targets: Analysis predicted that RES primarily targets cell survival, growth, and stress response pathways such as the PI3K/Akt/mTOR signaling pathway (PIK3CA, AKT1, mTOR), epithelial–mesenchymal transition (EMT)-related transcription factors (SNAI1, TWIST1), and regulators of the antioxidant defense system (Nrf2/KEAP1).

**Figure 16:**
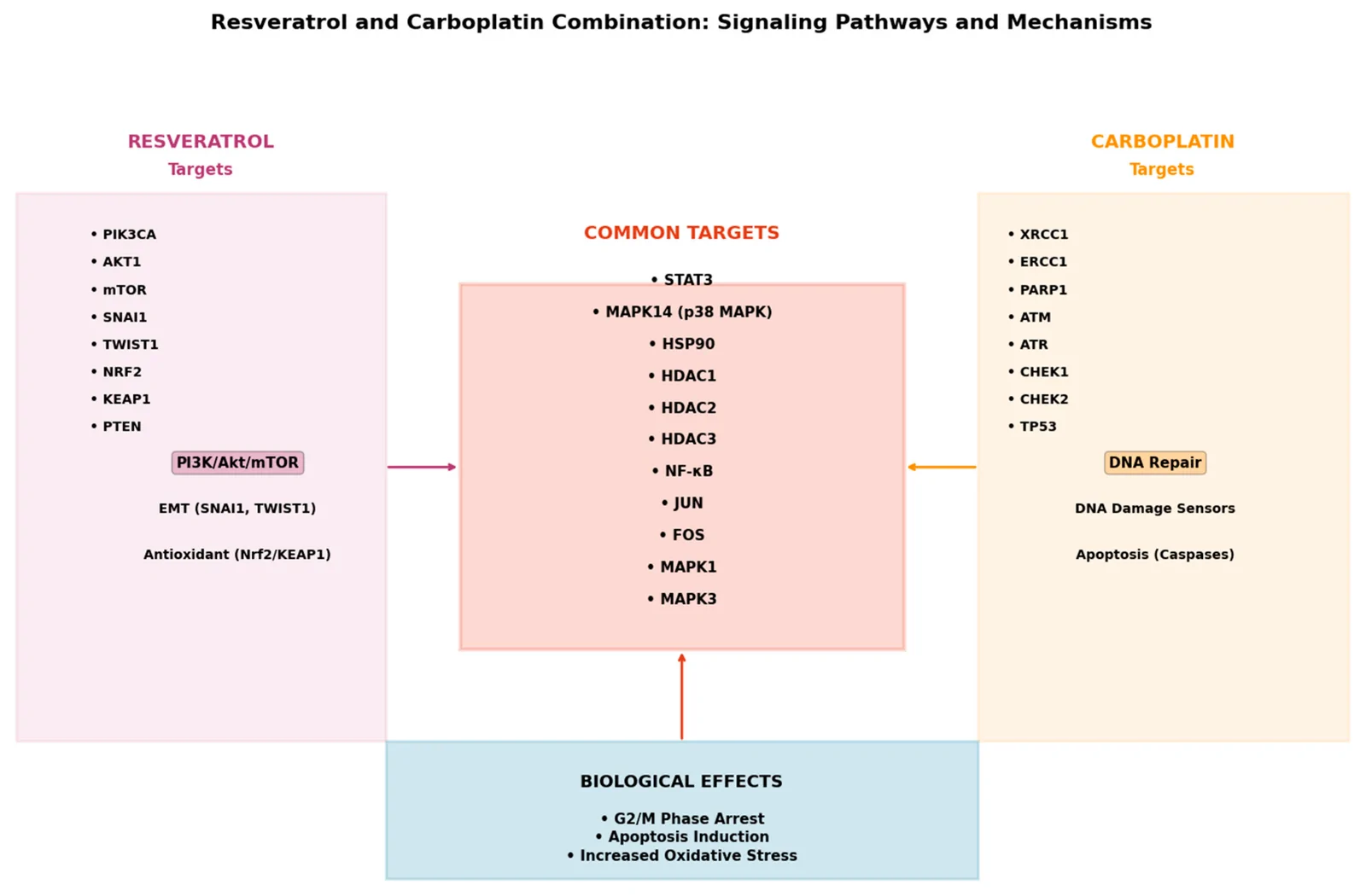
Schematic representation of potential signaling pathways associated with RES and CPT. The diagram illustrates predicted and literature-supported mechanisms of action and potential overlaps between RES and CPT. (**Left panel**) RES is associated with modulation of pathways including PI3K/Akt/mTOR signaling, EMT-related factors (SNAI1, TWIST1), and antioxidant response pathways (Nrf2/KEAP1). (**Right panel**) CPT is associated with DNA damage-related processes, including DNA repair machinery (XRCC1, ERCC1, PARP1), DNA damage sensors (ATM/ATR, CHEK1/2), and apoptotic pathways. (**Center panel**) Potential common targets, including STAT3, p38 MAPK, HSP90, and HDACs, may contribute to shared regulatory networks involved in cell survival and stress responses. (**Bottom panel**) These interactions are proposed to be associated with biological outcomes such as G2/M phase arrest, apoptosis, and increased oxidative stress.

CPT-Specific Targets: The highest-scoring targets for CPT were identified as proteins associated with DNA repair mechanisms (XRCC1, ERCC1, and PARP1), as well as cell cycle checkpoints (ATM, ATR, CHEK1, and CHEK2) and apoptotic signaling (CASP cascade and TP53), as expected. Overlapping Shared Targets: The intersection region of the Venn diagram showed critical targets, indicating the potential molecular basis for synergy between the two drugs. These shared targets include STAT3 (proliferation and survival signaling), MAPK14 (p38 MAPK) (stress response and apoptosis), HSP90 (client protein stabilization), and HDACs (histone deacetylases, epigenetic regulation). These targets suggest that both agents may disrupt the same signaling networks from different perspectives. These in silico findings are consistent with experimentally observed G2/M arrest, increased apoptosis, and oxidative stress, and they provide a mechanistic prediction that the combination may act through multiple interconnected pathways such as PI3K/Akt, DNA damage response, and epigenetic regulation (Figure 16).

**Figure 17:**
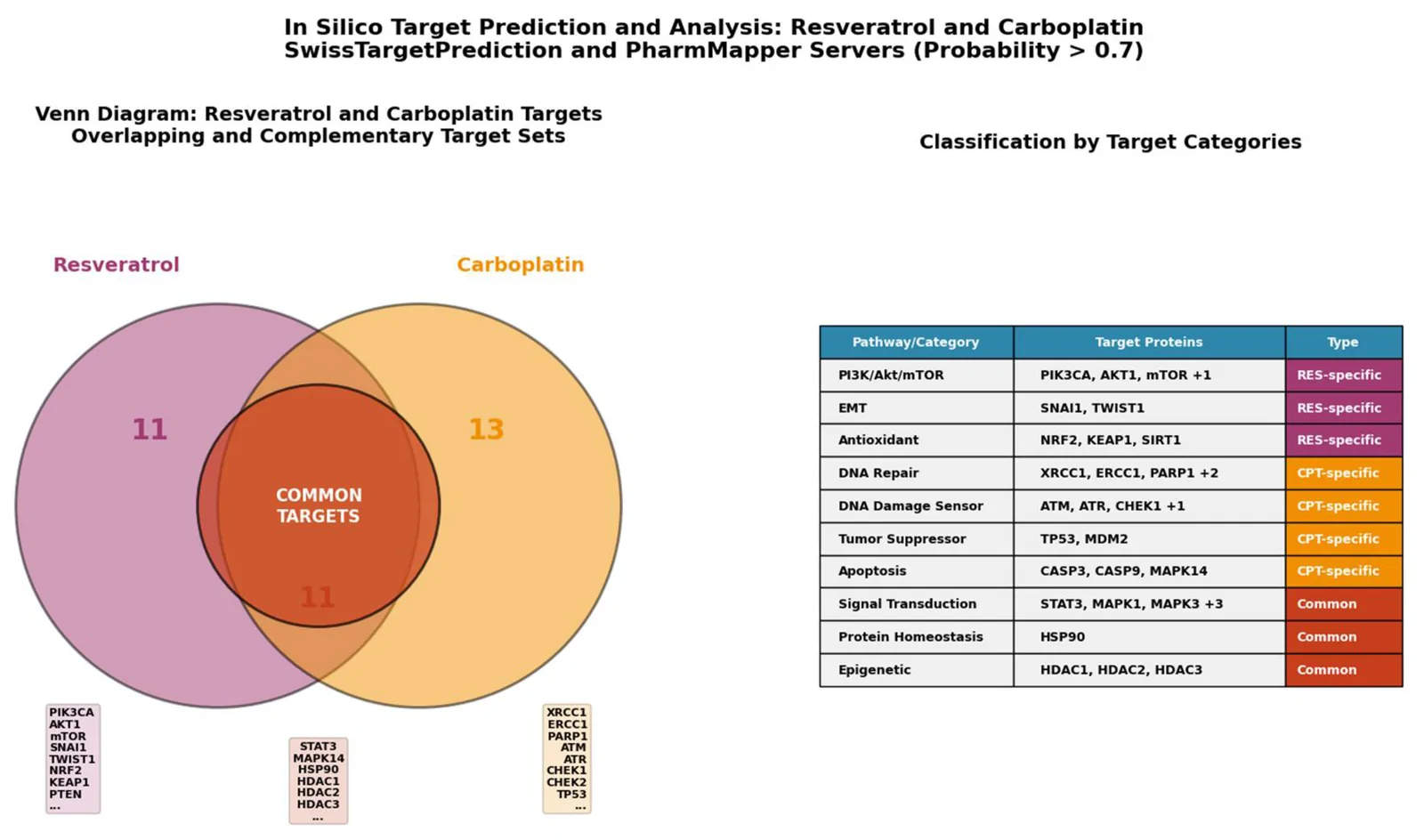
Venn diagram analysis of predicted molecular targets associated with RES and CPT. Target proteins were predicted using SwissTargetPrediction and PharmMapper servers with a probability threshold > 0.7. The diagram illustrates the distribution of RES-specific targets (purple, n = 11), CPT-specific targets (orange, n = 13), and common targets (red, n = 8). Example target lists are shown below each set. The overlapping region represents shared predicted targets between the two compounds, which may suggest potential overlap in their molecular interactions.

The most likely predicted biological targets (*p* > 0.7 probability cutoff) of both compounds were identified and compared. Venn diagram analysis revealed that RES and CPT have significantly overlapping and complementary target clusters (Figure 17).

## 3. Discussion

This study comprehensively evaluated the combined effects of RES and CPT in Y79 RB cells using complementary two-dimensional and three-dimensional experimental models. Our findings demonstrate that RES and CPT exert synergistic antiproliferative effects, as confirmed by CI analysis, and that this interaction is associated with enhanced apoptosis, G2/M phase cell cycle arrest, mitochondrial membrane depolarization, and increased intracellular ROS accumulation. Importantly, antioxidant rescue experiments revealed that NAC pretreatment significantly attenuated ROS levels and partially restored cell viability, supporting a contributory role of oxidative stress in mediating combination-induced cytotoxicity. At the molecular level, the combination modulated the transcription of apoptosis- and cell cycle-related genes and increased caspase-3 enzymatic activity, consistent with activation of the intrinsic apoptotic pathway. Furthermore, the enhanced antitumor efficacy of the combination was validated in a three-dimensional tumor spheroid model, where co-treatment markedly reduced spheroid size and viability compared with monotherapies. Collectively, these findings provide mechanistic and functional evidence supporting the therapeutic potential of this combination strategy in RB.

Our initial dose–response experiments revealed that both RES and CPT inhibit Y79 cell viability in a concentration and time-dependent manner. The IC_50_ values at 48 h were 46.1 µM for RES and 16.8 µM for CPT, indicating that CPT possesses greater cytotoxic potency in this cell line. These findings are consistent with previous studies reporting the antiproliferative effects of RES in various cancer cell lines, including RB [17,18]. CPT, as a platinum-based chemotherapeutic agent, exerts its cytotoxicity primarily through DNA crosslinking, leading to replication fork stalling and subsequent apoptotic cell death [8,19]. The time-dependent enhancement of cytotoxicity observed in our study, with lower IC_50_ values at 48 h compared to 24 h, aligns with the mechanism of action of both compounds, which require sufficient exposure time to trigger apoptotic cascades [20].

A key finding of this study is the demonstration of synergistic interaction between RES and CPT, validated using the Chou–Talalay CI method. All tested combinations yielded CI values less than 1, with the most pronounced synergy observed at the combination dose. This synergistic effect suggests that combining these agents may achieve enhanced efficacy at potentially lower concentrations, a strategy that could be relevant for reducing dose-dependent toxicity [21,22]. Similar synergistic interactions between RES and platinum-based compounds have been reported in other cancer models, including ovarian [23], lung [24], and colorectal cancer cells [25].

Flow cytometric analysis revealed that the RES + CPT combination significantly increased the apoptotic cell population (65.4%) compared to RES (24.5%) and CPT (31.2%) monotherapies. Annexin V/PI staining patterns indicated expansion of both early and late apoptotic populations, consistent with enhanced apoptotic cell death. Concurrently, cell cycle analysis demonstrated a marked accumulation of cells in the G2/M phase in the combination group compared with the control, indicating effective blockade of cell cycle progression at the G2/M checkpoint. G2/M arrest is a common response to DNA damage, allowing cells to repair lesions before entering mitosis; however, when damage is overwhelming, prolonged arrest can trigger apoptosis [26]. The observed G2/M arrest is consistent with CPT’s DNA-damaging mechanism and with reports that RES can interfere with cell cycle progression [27]. The enhanced effect of the combination on cell cycle arrest may reflect the convergence of DNA damage signals from CPT with the cell cycle regulatory effects of RES.

JC-1 staining revealed that the RES + CPT combination induced a 72% reduction in the red/green fluorescence ratio compared to the control, indicating severe mitochondrial membrane potential loss. This mitochondrial depolarization was significantly more pronounced than in monotherapy groups, suggesting enhanced disruption of mitochondrial integrity. Mitochondrial membrane potential loss is a critical early event in the intrinsic apoptotic pathway, leading to cytochrome c release and subsequent caspase activation [28].

Parallel assessment of intracellular ROS levels using DCFH-DA staining revealed a 3.5-fold increase in ROS production in the combination group. To determine whether this ROS increase plays a causal role in apoptosis induction, a rescue experiment was performed using the antioxidant NAC. NAC co-treatment significantly attenuated ROS levels (from 3.5-fold to 1.3-fold; *p* < 0.001) and partially restored cell viability in RES + CPT-treated cells. These findings indicate that ROS production plays a contributory role in the apoptotic effects induced by the RES + CPT combination. This aligns with reports that RES can exhibit pro-oxidant activities at higher concentrations [29,30] and that CPT generates ROS through its DNA-damaging effects and mitochondrial interactions [31]. The combination appears to exploit both mechanisms, resulting in amplified oxidative stress that contributes to mitochondrial dysfunction and cell death.

RT-qPCR analysis revealed that the RES + CPT combination significantly modulated the expression of key apoptosis- and cell cycle-related genes at the transcriptional level. Pro-apoptotic *BAX* expression was upregulated, while anti-apoptotic BCL-2 was downregulated, resulting in a 9.5-fold increase in the *BAX*/*BCL-2* mRNA ratio. This ratio is a critical determinant of cellular susceptibility to mitochondrial apoptosis, with higher values favoring mitochondrial outer membrane permeabilization [32]. The elevation of this ratio in combination-treated cells provides a transcriptional explanation for the enhanced mitochondrial apoptosis observed. Additionally, *CASP9* and *CASP3* mRNA expression were upregulated (3.5-fold and 3.8-fold, respectively). To confirm that increased mRNA expression translates to functional activation, caspase activity assays were performed. The combination treatment significantly increased caspase-3 activity (3.8-fold; *p* < 0.001), confirming activation of the effector caspase cascade at the functional level. Concurrently, cell cycle regulators *CCNB1* (cyclin B1) and *CDK1* were downregulated (0.35-fold and 0.32-fold, respectively), consistent with the observed G2/M arrest. These transcriptional changes correlate with the functional parameters (cell cycle distribution, apoptosis) and provide a mechanistic link between gene expression modulation and cellular outcomes.

In silico target prediction using SwissTargetPrediction and PharmMapper was performed to generate hypotheses regarding potential molecular targets of RES and CPT. RES was predicted to target proteins involved in survival signaling (PI3K/Akt/mTOR pathway components), stress responses (Nrf2/KEAP1), and epigenetic regulation (HDACs). CPT’s predicted targets centered on DNA repair machinery (XRCC1, ERCC1, and PARP1), DNA damage sensors (ATM, ATR, and CHEK1/2), and apoptotic caspases. The identification of common predicted targets, including STAT3, p38 MAPK, HSP90, and HDACs, is intriguing, as these proteins integrate multiple signaling pathways relevant to cancer cell survival and proliferation [33,34,35,36,37,38]. It is important to emphasize that these in silico predictions are hypothesis-generating only and require experimental validation. The predicted involvement of pathways such as PI3K/Akt, STAT3, and p38 MAPK was not experimentally tested in the current study. Future studies employing targeted inhibitors, Western blot analysis of phosphorylated proteins, or gene silencing approaches are necessary to determine whether these pathways contribute to the observed synergistic effects.

Immunocytochemical analysis of β-tubulin organization provided additional morphological evidence of enhanced cytotoxic effects in the combination group. While RES and CPT individually induced moderate cytoskeletal alterations, their combination caused marked disassembly and fragmentation of the microtubule network, accompanied by nuclear condensation and cellular fragmentation characteristic of apoptosis. The microtubule cytoskeleton plays essential roles in cell division, intracellular transport, and maintenance of cell shape; its disruption can trigger apoptosis through multiple mechanisms [38]. The enhanced cytoskeletal damage observed with the combination may result from a combined effect: CPT-induced DNA damage activates stress pathways that impinge on cytoskeletal organization, while RES’s reported ability interferes with microtubule dynamics [39]. This cytoskeletal disruption likely contributes to the observed G2/M arrest, as intact microtubules are essential for mitotic spindle formation and proper chromosome segregation.

The synergistic anticancer effects demonstrated in this study have important clinical implications for RB treatment. Current chemotherapeutic regimens for RB, while effective, are associated with significant toxicities, including myelosuppression, ototoxicity, and secondary malignancies [40]. The ability to achieve enhanced therapeutic efficacy with potentially lower doses of CPT through combination with RES could reduce dose-dependent toxicities while maintaining or improving clinical outcomes. RES’s favorable safety profile in humans, supported by numerous clinical trials for various conditions [4], makes it an attractive candidate for combination therapy.

Several limitations of this study should be acknowledged. First, all experiments were conducted using a single RB cell line (Y79), which may limit the generalizability of the findings to other RB subtypes. Future studies incorporating additional cell lines are necessary to confirm reproducibility. Second, the absence of a normal retinal cell model represents a significant limitation of the present study. All experiments were conducted exclusively in Y79 cancer cells, precluding direct assessment of whether the observed cytotoxic and synergistic effects are selective for malignant cells or may also affect non-malignant retinal cells. This limitation restricts the evaluation of the therapeutic index and safety profile of the RES + CPT combination, which is a critical parameter for translational relevance in anticancer combination studies [22]. Therefore, the clinical applicability of these findings remains uncertain and depends on future studies incorporating normal retinal cell models (e.g., ARPE-19) to determine cancer selectivity. Although transcriptional modulation of apoptosis- and cell cycle-related genes was demonstrated and caspase-3 activity was assessed functionally, quantitative protein-level validation of key mechanistic markers (e.g., BAX, BCL-2, cleaved caspase-3, PARP, cyclin B1, and CDK1) was not performed, which would strengthen mechanistic interpretation. In particular, the absence of protein-level and post-translational analyses (such as caspase cleavage or CDK1 phosphorylation status) limits definitive confirmation of pathway activation at the protein level. Nevertheless, the concordance between gene expression data and functional assays (apoptosis, caspase activity, and mitochondrial depolarization) provides supportive—though not definitive—evidence for activation of the intrinsic apoptotic pathway and G2/M arrest. Future studies incorporating Western blot or related proteomic approaches will be necessary to validate these mechanisms at the protein level. Moreover, despite CPT being a DNA-damaging agent, specific DNA damage markers such as γH2AX were not evaluated, limiting direct confirmation of DNA damage-mediated G2/M arrest. The in silico target predictions presented are hypothesis-generating and require experimental validation through targeted molecular approaches. Finally, the study was conducted entirely in vitro; therefore, the efficacy, safety profile, and pharmacokinetic properties of the RES + CPT combination must be evaluated in appropriate in vivo RB models before considering clinical translation.

## 4. Materials and Methods

### 4.1. Chemicals and Reagents

All chemicals used in this study were of analytical purity. RES (≥99% purity) and CPT were obtained from Sigma-Aldrich (St. Louis, MO, USA). RES was prepared as a 100 mM stock solution in dimethyl sulfoxide (DMSO) and stored at −20 °C until use. The final DMSO concentration in the cell culture medium was adjusted to not exceed 0.1%, and this concentration was confirmed to be non-cytotoxic to Y79 cells by preliminary tests. CPT was freshly dissolved in phosphate-buffered saline (PBS) prior to the experiment. RPMI-1640 medium, fetal bovine serum (FBS), penicillin–streptomycin mixture, and trypsin–EDTA solutions for cell culture were provided by Thermo Fisher Scientific (Waltham, MA, USA). Commercial kits used for apoptosis, cell cycle, ROS production, and mitochondrial membrane potential analyses were obtained from BD Biosciences (San Jose, CA, USA). β-tubulin antibody was used for immunocytochemical evaluation.

### 4.2. Culture Conditions of the Human Y79 Retinoblastoma Cell Line

Human RB cell line Y79 (ATCC^®^ HTB-18™) was cultured under standard suspension culture conditions. Cells were incubated in RPMI-1640 medium containing a mixture of 10% heat-inactivated FBS and 1% antibiotic at 37 °C in a humidified incubator containing 5% CO_2_. Cell density was maintained in the logarithmic growth phase by passage every 3–4 days, and all experimental procedures were performed in this phase.

### 4.3. Experimental Design and Drug Treatment Protocol

Prior to drug exposure, cells were seeded into culture plates and left for 24 h to allow stabilization. The experimental design comprised five distinct groups:

Control Group: Cells treated with culture medium containing 0.1% DMSO (carrier control).

RES Monotherapy Group: Cells treated with three different concentrations (5, 10, 25, 50, 75 and 100 µM) of RES for 24 and 48 h.

CPT Monotherapy Group: Cells treated with CPT at three different concentrations (2.5, 5, 10, 20, 30 and 40 µM) for 24 and 48 h.

Combination Therapy Group: Cells treated with concomitant combinations of RES and CPT at the indicated concentrations. Combination ratios were determined based on half maximal inhibitory concentration (IC_50_) values obtained from monotherapy dose–response curves.

ROS Suppression (Rescue) Group: To investigate whether ROS generation mediates the observed apoptotic effects, cells were co-treated with the RES + CPT combination and the antioxidant NAC (NAC, 5 mM).

For other analyses in the study (e.g., ROS, qRT-PCR, and flow cytometry), RES50 and CPT20 doses were used because these doses were closest to the 48 h IC_50_ value.

### 4.4. Cell Viability Assessment by MTT Assay

Antiproliferative effects were evaluated using the MTT method. Y79 cells were seeded into 96-well plates and exposed to the indicated treatments after 24 h of incubation. At the end of the treatment period, MTT reagent was added and incubated for 4 h. The resulting formazan crystals were dissolved with DMSO, and the absorbance was measured at 570 nm. Cell viability was calculated as a percentage compared to the control group. IC_50_ values were determined using nonlinear regression analysis. IC_50_ values were derived through nonlinear regression analysis employing GraphPad Prism 9.0 software.

### 4.5. Drug Interaction and Synergy Analysis (CI and Bliss Model)

Drug interactions were calculated using CI analysis based on the Chou–Talalay method. CI < 1 indicates synergy, CI = 1 indicates an additive effect, and CI > 1 indicates antagonism. Calculations were performed using specialized software. Additionally, the results were validated using the Bliss independence model.

For additional validation of drug interaction, the Bliss independence model was applied. The expected additive effect (E_Bliss) for combination treatments was calculated according to the formula:

E_Bliss = A + B − (A × B),

where A and B represent the fractional inhibition values produced by every single agent. Percentage inhibition values were converted to fractional values prior to calculation. A Bliss excess value greater than zero (observed effect − expected effect > 0) was interpreted as a synergistic interaction.

### 4.6. Flow Cytometric Analysis of Apoptosis (Annexin V-FITC/PI Assay)

The percentage of apoptotic cells was determined by Annexin V-FITC/PI double staining. After treatment, cells were suspended in binding buffer, stained, and analyzed by flow cytometry. Early and late apoptosis percentages were calculated separately. Data analysis was conducted with FlowJo software (FlowJo LLC, Ashland, OR, USA; available at https://www.flowjo.com, accessed on 2 February 2026).

### 4.7. Flow Cytometric Analysis of Cell Cycle Distribution (PI Staining)

Cells were fixed with ethanol and stained with PI after RNase application, and DNA content was measured by flow cytometry to determine the G0/G1, S, and G2/M phase distributions.

### 4.8. Assessment of Mitochondrial Membrane Potential (ΔΨm) by JC-1 Staining

The cationic dye JC-1 (5,5′,6,6′-tetrachloro-1,1′,3,3′-tetraethylbenzimidazolecarbocyanin iodide) was used to measure ΔΨm. ΔΨm assessment was performed using JC-1 dye. The red/green fluorescence ratio was calculated as an indicator of mitochondrial integrity. The ratio of red (aggregate) and green (monomer) fluorescence intensities was calculated as an indicator of mitochondrial health.

### 4.9. Flow Cytometric Measurement of Intracellular ROS (DCFH-DA Assay)

Intracellular ROS levels were determined using the fluorescent probe 2′,7′-dichlorodihydrofluorescein diacetate (DCFH-DA). Post-treatment cells were incubated with 10 µM DCFH-DA for 30 min at 37 °C. Cells were washed and resuspended in PBS, and the mean fluorescence intensity (MFI) of DCF (excitation/emission: 488/525 nm) was measured by flow cytometry.

### 4.10. Evaluation of ROS-Dependent Cytotoxicity by NAC Pretreatment

To investigate whether oxidative stress contributes functionally to the cytotoxic effects of RES and CPT, a rescue experiment was performed using the antioxidant NAC.

Y79 cells were pretreated with NAC (5 mM) for 2 h at 37 °C prior to drug exposure. Following the 2 h pretreatment period, NAC was maintained in the culture medium, and cells were subsequently exposed to RES (50 µM), CPT (20 µM), or their combination (RES 50 µM + CPT 20 µM) for an additional 48 h in the continuous presence of NAC.

The following experimental groups were included:

- Control (0.1% DMSO)
- NAC alone (5 mM)
- RES (50 µM)
- CPT (20 µM)
- RES + CPT
- RES + NAC
- CPT + NAC
- RES + CPT + NAC

This design allowed assessment of whether ROS suppression selectively modulates combination-induced cytotoxicity or also affects monotherapy responses.

#### 4.10.1. Intracellular ROS Measurement Following NAC Pretreatment

Intracellular ROS levels were quantified using DCFH-DA. After 48 h of treatment, cells were incubated with 10 µM DCFH-DA at 37 °C for 30 min in the dark. Cells were then washed twice with PBS and analyzed immediately by flow cytometry (excitation 488 nm; emission 525 nm).

A total of 20,000 events were acquired per sample. MFI values were calculated using FlowJo software and expressed as fold change relative to the control group.

#### 4.10.2. Cell Viability Assessment by MTT Assay After NAC Pretreatment

To evaluate whether ROS suppression influenced cytotoxicity, cell viability was assessed using the MTT assay following NAC pretreatment.

After the 48 h treatment period, MTT reagent (final concentration 0.5 mg/mL) was added to each well and incubated for 4 h at 37 °C. Formazan crystals were dissolved in DMSO, and absorbance was measured at 570 nm using a microplate reader. Cell viability was calculated as a percentage relative to the control group.

All experiments were performed in three independent biological replicates with at least three technical replicates per condition.

### 4.11. Colorimetric Assay for Caspase-3 Activity

To evaluate the functional role of the caspase cascade in combination therapy-induced apoptosis, caspase-3 enzyme activity was measured using a colorimetric method. Y79 cells were treated with RES, CPT, and a combination thereof for 48 h. Caspase-3 activity measurement was performed using the Caspase-3 Colorimetric Activity Assay Kit (R&D Systems, Minneapolis, MN, USA) according to the manufacturer’s protocol.

### 4.12. Quantitative Real-Time PCR (RT-qPCR) Analysis

Gene expression analysis was conducted using quantitative reverse transcription PCR (RT-qPCR). Total RNA isolation was followed by cDNA synthesis. Expression levels of target genes were analyzed using SYBR Green-based RT-qPCR. GAPDH and ACTB were used as reference genes for normalization, and relative expression levels were calculated using the 2^−ΔΔCt^ method based on the geometric mean of their Ct values. All samples were run in triplicate, and no-template controls were included in each run to verify the absence of contamination. Specific primer sequences are listed in Table 1 (synthesized and purified by HPLC).

Primer specificity was validated for each gene using dissociation curve analysis, and all primer pairs generated a single specific peak. Data were analyzed using mean Ct (threshold cycle) values. GAPDH and ACTB were used as reference genes for normalization, and relative expression levels were calculated using the 2^−ΔΔCt^ method based on the geometric mean of their Ct values. Data were expressed as fold change relative to the control group.

### 4.13. Immunocytochemical Evaluation of β-Tubulin Organization

Y79 RB cells were cultured under standard conditions and seeded onto poly-L-lysine-coated glass coverslips to facilitate cell attachment. Following treatment with RES (50 µM), CPT (20 µM), or their combination for 48 h, cells were collected and fixed. After fixation, cells were permeabilized with 0.1% Triton X-100 in PBS for 10 min. Non-specific binding sites were blocked using 5% bovine serum albumin in PBS for 1 h. Cells were then incubated overnight at 4 °C with an anti-β-tubulin primary antibody (Sigma-Aldrich, St. Louis, MO, USA). After cells were incubated with an appropriate secondary antibody (Alexa Fluor 488-conjugated) for 1 h. Nuclei were counterstained with 4′,6-Diamidino-2-Phenylindole (DAPI) (1 µg/mL). All fluorescence images were captured using a Zeiss Axio Cam fluorescence microscope (Carl Zeiss AG, Oberkochen, Germany).

This analysis was not performed to measure quantitative protein expression levels, but rather to visualize morphological changes in cytoskeletal organization and to qualitatively confirm apoptotic morphology.

### 4.14. Three-Dimensional Tumor Spheroid Culture and Analysis

#### 4.14.1. Formation of 3D Y79 Tumor Spheroids

To evaluate the effects of RES and CPT in a three-dimensional tumor model, Y79 RB spheroids were generated using ultra-low attachment (ULA) 96-well round-bottom plates (Corning Inc., Corning, NY, USA). This system was employed to better mimic tumor-like cell–cell interactions and spatial architecture compared to conventional suspension culture.

Exponentially growing Y79 cells were collected and resuspended in complete RPMI-1640 medium supplemented with 10% FBS and 1% penicillin–streptomycin. Cells were seeded at a density of 3 × 10^3^ cells per well in a final volume of 200 µL and incubated at 37 °C in a humidified atmosphere containing 5% CO_2_.

Compact and uniformly shaped spheroids formed within 48–72 h, as confirmed by inverted light microscopy. Only spheroids with a single, well-defined spherical morphology were included in subsequent analyses.

#### 4.14.2. Drug Treatment of 3D Spheroids

After spheroid formation, the culture medium was carefully replaced with fresh medium containing RES (50 µM), CPT (20 µM), or their combination (RES 50 µM + CPT 20 µM). These concentrations correspond to the IC_50_ values determined in 2D experiments at 48 h.

The following experimental groups were established:

- Control (0.1% DMSO);
- RES (50 µM);
- CPT (20 µM);
- RES + CPT.

Spheroids were incubated with treatments for 72 h. Vehicle concentration was kept constant across all groups, and treatment-containing medium was not replaced during the incubation period.

#### 4.14.3. Bright-Field Imaging and Spheroid Size Quantification

Bright-field images were acquired using an Olympus IX73 inverted microscope equipped with a digital camera at 10× magnification (Olympus Corporation, Tokyo, Japan). Images were captured using identical optical settings for all experimental groups.

Spheroid diameter was measured using ImageJ software (version 1.54; National Institutes of Health, Bethesda, MD, USA). For each condition, at least five spheroids per group from three independent experiments were analyzed. Mean spheroid diameter values were calculated and expressed as mean ± SD.

#### 4.14.4. Cell Viability Assessment in 3D Spheroids

Cell viability within 3D spheroids was assessed using the ATP-based CellTiter-Glo^®^ 3D Cell Viability Assay (Promega, Madison, WI, USA), according to the manufacturer’s instructions. An equal volume of CellTiter-Glo^®^ 3D reagent was added directly to each well and incubated for 30 min at room temperature with gentle shaking to ensure complete spheroid lysis.

Luminescence was measured using a microplate reader (BioTek, Winooski, VT, USA). Viability values were normalized to the control group and expressed as a percentage of the control.

#### 4.14.5. Live/Dead Fluorescence Staining and Image Acquisition

Live/dead staining of 3D Y79 tumor spheroids was performed using Calcein-AM (2 µM) and Ethidium homodimer-1 (4 µM). After 72 h of treatment, spheroids were incubated with staining solution for 30 min at room temperature in the dark.

Fluorescence images were acquired using an inverted fluorescence microscope equipped with FITC and TRITC filter sets at 10× magnification. Images were captured using identical exposure conditions for all experimental groups. Images were obtained at the spheroid mid-plane using a single focal plane to ensure consistency.

### 4.15. Statistical Analysis

Data were obtained from at least three independent biological experiments (n = 3), each performed with a minimum of three technical replicates where applicable. Results are presented as mean ± standard deviation (SD). Normality of data distribution was assessed using the Shapiro–Wilk test prior to statistical comparisons.

For comparisons among multiple groups, one-way analysis of variance (ANOVA) followed by Tukey’s post hoc multiple comparison test was performed. For experiments involving multiple conditions across a single factor (e.g., treatment groups), statistical comparisons were conducted relative to the control group unless otherwise specified. For selected analyses involving multiple pairwise comparisons (e.g., caspase-3 activity and spheroid assays), significance is indicated between groups as shown in the respective figures.

IC_50_ values were calculated using nonlinear regression analysis (log[inhibitor] vs. normalized response, variable slope model).

CI values were calculated according to the Chou–Talalay method using mean values derived from three independent experiments. Bliss independence analysis was performed using the formula E_bliss = A + B − (A × B), where A and B represent fractional inhibition values of single treatments. Bliss excess values greater than zero were interpreted as indicative of potential synergistic interaction. However, CI and Bliss analyses were based on mean values without incorporation of variability measures (e.g., standard deviation or confidence intervals) and should therefore be interpreted descriptively.

For flow cytometry and fluorescence-based assays (e.g., apoptosis, ROS, and mitochondrial membrane potential), representative plots or histograms are shown, while quantitative analyses are derived from three independent experiments.

All statistical analyses and graphical representations were generated using GraphPad Prism version 9.0 (GraphPad Software, San Diego, CA, USA). A *p*-value < 0.05 was considered statistically significant. Statistical significance levels were defined as follows: * *p* < 0.05, ** *p* < 0.01, and *** *p* < 0.001.

## 5. Conclusions

In summary, the present study demonstrates that the combination of RES and CPT exerts a synergistic antiproliferative effect in Y79 RB cells, as confirmed by CI and Bliss analyses. This enhanced cytotoxic interaction is associated with increased apoptotic cell death, marked G2/M phase arrest, mitochondrial membrane depolarization, and elevated intracellular ROS levels. Antioxidant rescue experiments revealed that NAC pretreatment significantly attenuated ROS accumulation and partially restored cell viability, supporting a contributory role of oxidative stress in mediating combination-induced cytotoxicity. At the molecular level, the co-treatment increased the *BAX*/*BCL-2* ratio and upregulated *CASP3* and *CASP9* expression while downregulating *CCNB1* and *CDK1*, in parallel with increased caspase-3 enzymatic activity, collectively consistent with activation of the intrinsic apoptotic pathway and cell cycle blockade. Importantly, the enhanced antitumor efficacy of the combination was preserved in a three-dimensional tumor spheroid model, where co-treatment significantly reduced spheroid size and viability and increased cell death compared with monotherapies.

Taken together, these findings provide integrated functional and transcriptional evidence that RES may potentiate CPT-induced cytotoxicity through coordinated modulation of oxidative stress, mitochondrial integrity, and cell cycle regulation. Nevertheless, given the in vitro nature of the study and the absence of normal retinal cells and in vivo validation, these results should be considered preclinical and hypothesis-generating. Further investigation in additional RB models and appropriate in vivo systems is required to determine translational relevance and therapeutic selectivity.

## Figures and Tables

**Table 1:** Primer sequences used for qRT-PCR.

TARGET GENE	Forward Primer (5′→3′)	Reverse Primer (5′→3′)
*BAX*	TCAGGATGCGTCCACCAAGAAG	TGTGTCCACGGCGGCAATCATC
*BCL2*	ATCGCCCTGTGGATGACTGAGT	GCCAGGAGAAATCAAACAGAGGC
*CASP3*	AGAGGGGATCGTTGTAGAAGCTG	CACAAGCGACTGGATGAACCA
*CASP9*	CCTCATCATCAACAACCTGG	AAGTCCCTTTCGCAGAAACAG
*CCNB1*	CCGTCCATGCGGAAGATC	ATGGCCAGCGGGAAGAC
*CDK1*	GGAAACCAGGAAGCCTAGCATC	GGATGATTCAGTGCCATTTTGCC
*ACTB*	CATTGCTGACAGGATGCAGAAGG	TGCTGGAAGGTGGACAGTGAGG
*GAPDH*	GGAGCGAGATCCCTCCAAAAT	GGCTGTTGTCATACTTCTCATGG

## Data Availability

The original contributions presented in this study are included in the article. Further inquiries can be directed to the corresponding author.

## Abbreviations

ACTB	Actin Beta (reference gene)
AKT	Protein Kinase B
ANOVA	Analysis of Variance
ATM	Ataxia Telangiectasia Mutated
ATR	Ataxia Telangiectasia and Rad3-Related
BAX	BCL-2-Associated X Protein
BCL-2	B-Cell Lymphoma 2
CCNB1	Cyclin B1
CDK1	Cyclin-Dependent Kinase 1
CHEK1/2	Checkpoint Kinase 1/2
CI	Combination Index
CO_2_	Carbon Dioxide
CPT	Carboplatin
Ct	Threshold Cycle
DCFH-DA	2′,7′-Dichlorodihydrofluorescein Diacetate
DAPI	4′,6-Diamidino-2-Phenylindole
DNA	Deoxyribonucleic Acid
DMSO	Dimethyl Sulfoxide
EMT	Epithelial–Mesenchymal Transition
ERCC1	Excision Repair Cross-Complementation Group 1
Fa	Fraction Affected
FBS	Fetal Bovine Serum
FITC	Fluorescein Isothiocyanate
GAPDH	Glyceraldehyde-3-Phosphate Dehydrogenase
G2/M	Gap 2/Mitosis Phase
HDAC	Histone Deacetylase
HSP90	Heat Shock Protein 90
IC_50_	Half Maximal Inhibitory Concentration
KEAP1	Kelch-Like ECH-Associated Protein 1
MAPK14	Mitogen-Activated Protein Kinase 14 (p38 MAPK)
MFI	Mean Fluorescence Intensity
MTT	3-(4,5-Dimethylthiazol-2-yl)-2,5-Diphenyltetrazolium Bromide
mTOR	Mammalian Target of Rapamycin
NAC	N-Acetylcysteine
Nrf2	Nuclear Factor Erythroid 2-Related Factor 2
PARP1	Poly(ADP-Ribose) Polymerase 1
PBS	Phosphate-Buffered Saline
PI	Propidium Iodide
PI3K	Phosphoinositide 3-Kinase
RB	Retinoblastoma
RB1	Retinoblastoma 1 Gene
RES	Resveratrol
RNA	Ribonucleic Acid
ROS	Reactive Oxygen Species
RT-qPCR	Quantitative Reverse Transcription Polymerase Chain Reaction
SD	Standard Deviation
STAT3	Signal Transducer and Activator of Transcription 3
TP53	Tumor Protein p53
TRITC	Tetramethylrhodamine Isothiocyanate
ULA	Ultra-Low Attachment
XRCC1	X-Ray Repair Cross-Complementing Protein 1
ΔΨm	Mitochondrial Membrane Potential

## References

[1] Dimaras H., Kimani K., Dimba E.A., Gronsdahl P., White A., Chan H.S., Gallie B.L. (2012). Retinoblastoma. Lancet.

[2] Abramson D.H., Shields C.L., Munier F.L., Chantada G.L. (2015). Treatment of retinoblastoma in 2015: Agreement and disagreement. JAMA Ophthalmol..

[3] Yousef Y.A., Soliman S.E., Astudillo P.P.P., Durairaj P., Dimaras H., Chan H.S.L., Heon E., Gallie B.L. (2016). Intra-arterial chemotherapy for retinoblastoma: A systematic review. JAMA Ophthalmol..

[4] Rodriguez-Galindo C., Orbach D.B., VanderVeen D. (2015). Retinoblastoma. Pediatr. Clin. N. Am..

[5] Özdemir İ., Afşin Y., Tuncer M.C., Öztürk Ş. (2025). Combined hesperidin and doxorubicin treatment induces apoptosis and modulates inflammatory cytokines in HeLa cervical cancer cells. Int. J. Mol. Sci..

[6] Samuel V.P., Gupta G., Dahiya R., Jain D.A., Mishra A., Dua K. (2019). Current update on preclinical and clinical studies of resveratrol, a naturally occurring phenolic compound. Crit. Rev. Eukaryot. Gene Expr..

[7] Singh A.P., Singh R., Verma S.S., Rai V., Kaschula C.H., Maiti P., Gupta S.C. (2019). Health benefits of resveratrol: Evidence from clinical studies. Med. Res. Rev..

[8] Ko J.H., Sethi G., Um J.Y., Shanmugam M.K., Arfuso F., Kumar A.P., Bishayee A., Ahn K.S. (2017). The Role of Resveratrol in Cancer Therapy. Int. J. Mol. Sci..

[9] Cakina S., İrkin L.C., Öztürk Ş. (2024). Protective effects of curcumin and resveratrol on kidney tissue on cadmium-induced oxidative stress in rats. Gazi Med. J..

[10] Pervaiz S., Holme A.L. (2009). Resveratrol: Its biologic targets and functional activity. Antioxid. Redox Signal..

[11] Kelland L. (2007). The resurgence of platinum-based cancer chemotherapy. Nat. Rev. Cancer.

[12] Ghosh S. (2019). Cisplatin: The first metal based anticancer drug. Bioorg. Chem..

[13] Li W., Cao L., Chen X., Lei J., Ma Q. (2016). Resveratrol inhibits hypoxia-driven ROS-induced invasive and migratory ability of pancreatic cancer cells via suppression of the Hedgehog signaling pathway. Oncol. Rep..

[14] Yin H., Wang Y., Chen W., Zhong S., Liu Z., Zhao J. (2016). Drug-resistant CXCR4-positive cells have the molecular characteristics of EMT in NSCLC. Gene.

[15] Annaji M., Poudel I., Boddu S.H.S., Arnold R.D., Tiwari A.K., Babu R.J. (2021). Resveratrol-loaded nanomedicines for cancer applications. Cancer Rep..

[16] Yang M.-D., Sun Y., Zhou W.-J., Xie X.-Z., Zhou Q.-M., Lu Y.-Y., Su S.-B. (2021). Resveratrol Enhances Inhibition Effects of Cisplatin on Cell Migration and Invasion and Tumor Growth in Breast Cancer MDA-MB-231 Cell Models In Vivo and In Vitro. Molecules.

[17] Sareen D., van Ginkel P.R., Takach J.C., Mohiuddin A., Darjatmoko S.R., Albert D.M., Polans A.S. (2006). Mitochondria as the primary target of resveratrol-induced apoptosis in human retinoblastoma cells. Investig. Ophthalmol. Vis. Sci..

[18] van Ginkel P.R., Sareen D., Subramanian L., Walker Q., Darjatmoko S.R., Lindstrom M.J., Kulkarni A., Albert D.M., Polans A.S. (2007). Resveratrol inhibits tumor growth of human neuroblastoma and mediates apoptosis by directly targeting mitochondria. Clin. Cancer Res..

[19] Wang D., Lippard S.J. (2005). Cellular processing of platinum anticancer drugs. Nat. Rev. Drug Discov..

[20] Siddik Z.H. (2003). Cisplatin: Mode of cytotoxic action and molecular basis of resistance. Oncogene.

[21] Chou T.C. (2006). Theoretical basis, experimental design, and computerized simulation of synergism and antagonism in drug combination studies. Pharmacol. Rev..

[22] Foucquier J., Guedj M. (2015). Analysis of drug combinations: Current methodological landscape. Pharmacol. Res. Perspect..

[23] Nessa M.U., Beale P., Chan C., Yu J.Q., Huq F. (2011). Synergism from combinations of cisplatin and oxaliplatin with quercetin and thymoquinone in human ovarian tumour models. Anticancer Res..

[24] Moar K., Brahma M., Pant A., Maruthi M., Maurya P.K. (2024). Synergistic Anticancer Activity of Resveratrol with Cisplatin and Carboplatin in A549 Lung Adenocarcinoma Cells. Int. J. Clin. Exp. Pathol..

[25] Vernousfaderani E.K., Akhtari N., Rezaei S., Rezaee Y., Shiranirad S., Mashhadi M., Hashemi A., Khankandi H.P., Behzad S. (2021). Resveratrol and Colorectal Cancer: A Molecular Approach to Clinical Researches. Curr. Top. Med. Chem..

[26] Hankittichai P., Thaklaewphan P., Wikan N., Ruttanapattanakul J., Potikanond S., Smith D.R., Nimlamool W. (2023). Resveratrol enhances cytotoxic effects of cisplatin by inducing cell cycle arrest and apoptosis in ovarian adenocarcinoma SKOV-3 cells through activating the p38 MAPK and suppressing AKT. Pharmaceuticals.

[27] Liang Y.C., Tsai S.H., Chen L., Lin-Shiau S.Y., Lin J.K. (2003). Resveratrol-induced G2 arrest through the inhibition of CDK7 and p34CDC2 kinases in colon carcinoma HT-29 cells. Biochem. Pharmacol..

[28] Kroemer G., Galluzzi L., Brenner C. (2007). Mitochondrial membrane permeabilization in cell death. Physiol. Rev..

[29] Zorov D.B., Juhaszova M., Sollott S.J. (2014). Mitochondrial reactive oxygen species (ROS) and ROS-induced ROS release. Physiol. Rev..

[30] de la Lastra C.A., Villegas I. (2007). Resveratrol as an antioxidant and pro-oxidant agent: Mechanisms and clinical implications. Biochem. Soc. Trans..

[31] Marullo R., Werner E., Degtyareva N., Moore B., Altavilla G., Ramalingam S.S., Doetsch P.W. (2013). Cisplatin induces a mitochondrial-ROS response that contributes to cytotoxicity depending on mitochondrial redox status and bioenergetic functions. PLoS ONE.

[32] Birkinshaw R.W., Czabotar P.E. (2017). The BCL-2 family of proteins and mitochondrial outer membrane permeabilisation. Semin. Cell Dev. Biol..

[33] Berman A.Y., Motechin R.A., Wiesenfeld M.Y., Holz M.K. (2017). The therapeutic potential of resveratrol: A review of clinical trials. NPJ Precis. Oncol..

[34] Duman E., Maçin A., Özdemir İ., Öztürk Ş., Tuncer M.C. (2026). Synergistic Antitumor Effects of Rosmarinic Acid and Cisplatin in Retinoblastoma: Evidence from 2D and 3D Tumor Models. Biomedicines.

[35] Moser C., Ruemmele P., Gehmert S., Schenk H., Kreutz M.P., Mycielska M.E., Hackl C., Kroemer A., Schnitzbauer A.A., Stoeltzing O. (2012). STAT5b as molecular target in pancreatic cancer inhibition of tumor growth, angiogenesis, and metastases. Neoplasia.

[36] Duman E., Maçin A., Özdemir İ., Öztürk Ş., Tuncer M.C. (2026). Quercetin Sensitizes Retinoblastoma Cells to Mitomycin C Through Transcriptional Modulation of p53-Regulated Apoptotic Genes: A Preclinical Study. Pharmaceuticals.

[37] Whitesell L., Lindquist S.L. (2005). HSP90 and the chaperoning of cancer. Nat. Rev. Cancer.

[38] Bolden J.E., Peart M.J., Johnstone R.W. (2006). Anticancer activities of histone deacetylase inhibitors. Nat. Rev. Drug Discov..

[39] Jordan M.A., Wilson L. (2004). Microtubules as a target for anticancer drugs. Nat. Rev. Cancer.

[40] Chabert P., Fougerousse A., Brouillard R. (2006). Anti-mitotic properties of resveratrol analog (z)-3,5,4′-trimethoxystilbene. BioFactors.

