# Correction: Maçin et al. Synergistic Anticancer Effects of Resveratrol and Carboplatin in Y79 Retinoblastoma Cells: Mechanistic Insights into Apoptosis, G2/M Arrest, and ROS-Dependent Mitochondrial Dysfunction. *Int. J. Mol. Sci.* 2026, *27*, 3473

**DOI:** 10.3390/ijms27188402

**Published:** 2026-09-21

**Authors:** Aydın Maçin, Erkan Duman, İlhan Özdemir, Mehmet Cudi Tuncer

**Affiliations:** 1Department of Ophthalmology, Diyarbakır Private Batı Hospital, 21100 Diyarbakır, Turkey; draydinmacin@outlook.com; 2Department of Ophthalmology, WestEye Private Hospital, Erbil 44001, Iraq; ophthalmo48@outlook.com; 3Department of Histology and Embryology, Faculty of Medicine, Kahramanmaraş Sütçü İmam University, 46040 Kahramanmaraş, Turkey; ilhanozdemir25@yandex.com; 4Department of Anatomy, Faculty of Medicine, Dicle University, 21280 Diyarbakır, Turkey

## References

In the original publication [1], Reference [16] was retracted. The authors replaced the retracted reference with the following:

16. Yang, M.-D.; Sun, Y.; Zhou, W.-J.; Xie, X.-Z.; Zhou, Q.-M.; Lu, Y.-Y.; Su, S.-B. Resveratrol Enhances Inhibition Effects of Cisplatin on Cell Migration and Invasion and Tumor Growth in Breast Cancer MDA-MB-231 Cell Models In Vivo and In Vitro. *Molecules*
**2021**, *26*, 2204. https://doi.org/10.3390/molecules26082204.

The authors state that the scientific conclusions are unaffected. This correction was approved by the Academic Editor. The original publication has also been updated.
